# Identification of *Morus notabilis* MADS-box genes and elucidation of the roles of *MnMADS33* during endodormancy

**DOI:** 10.1038/s41598-018-23985-0

**Published:** 2018-04-11

**Authors:** Yiwei Luo, Hongshun Li, Zhonghuai Xiang, Ningjia He

**Affiliations:** State Key Laboratory of Silkworm Genome Biology, Southwest University, Beibei, Chongqing 400715 P. R. China

## Abstract

The MADS-box genes encode transcriptional regulators with various functions especially during floral development. A total of 54 putative *Morus notabilis* MADS-box genes (*MnMADS*s) were identified and phylogenetically classified as either type I (17 genes) or type II (37 genes). The detected genes included three *FLOWERING LOCUS C-*like (*MnFLC-*like) genes, *MnMADS33*, *MnMADS50*, and *MnMADS7*. MnFLC-like proteins could directly or indirectly repress promoter activity of the mulberry *FLOWERING LOCUS T*-like (*MnFT*) gene. Transgenic *Arabidopsis thaliana* overexpressing *MnFLC-*like genes exhibited delayed flowering and down-regulated expression of *FT* and *SUPPRESSOR OF OVEREXPRESSION OF CONSTANS1* (*SOC1*). The gene expression analyses in floral bud indicated that *MnMADS33* expression increased, while *MnFT* expression decreased during the induction of dormancy in response to cold conditions. Dormancy release resulted in the down-regulation of *MnMADS33* expression and the up-regulation of *MnFT* expression. Furthermore, abscisic acid promoted the transcription of *MnMADS33* and *MnFT*, although the expression level of *MnFT* gradually decreased. These results are consistent with the hypothesis that *MnMADS33* negatively regulated the expression of *MnFT* to repress dormancy release and flowering in mulberry. This study may be relevant for future investigations regarding the effects of *MnMADS* genes on mulberry flowering development.

## Introduction

The MADS-box gene family is not specific to plants as it is also commonly found in animals and fungi. The MADS-box genes, which encode transcription factors, are conserved among eukaryotes^1,2^. The DNA-binding domain at the N-terminus of these transcription factors consists of a conserved region (approximately 58 amino acids) called the MADS-box domain, which is also involved in dimerization reactions and the binding of accessory factors^3^. The CArG-box [C(A/T)8G, C(C/T)(A/T)G(A/T)4(A/G), or C(C/T)(A/T)6(A/G)G] is a consensus MADS-box transcription factor-binding site^4–6^. In the model plant *Arabidopsis thaliana*, there are two types of MADS-box genes that differ regarding their conserved sequences and structures. Type I MADS-box genes encode only the MADS-box domain, and are further divided into the Mα, Mβ, and Mγ subclasses. In addition to the MADS-box domain, type II genes encode the intervening (I), keratin-like (K), and C-terminal (C) domains that ultimately form the so-called MIKC-type domain structure^7,8^. The I and K domains affect protein-protein interactions, resulting in the formation of homodimeric or heterodimeric complexes, while the highly variable C domain influences transcriptional regulation and contributes to the formation of protein complexes^2,9^. Type II genes are classified into the MIKC^c^ and MIKC* subfamilies^7^. Based on their sequence characteristics and functions, the MIKC^c^ genes have been further divided into 13 subcategories, which were named after their first identified members^1,10,11^.

There is a limited amount of information regarding the *A*. *thaliana* type I MADS-box genes. In addition, the type II genes include the floral homeotic genes, whose functions are related to the determination of floral organ identities, which can be explained by the well-studied ABCDE model^12–14^. All ABCDE model genes belong to the MIKC^c^ subfamily, with the exception of *AP2*^15^. The *APETALA1*/*FRUITFULL* (*AP1*/*FUL*), *APETALA3*/*PISTILLATA* (*AP3*/*PI*), *AGAMOUS* (*AG*), *SHATTERPROOF*/*SEEDSTICK* (*SHP*/*STK*), and *SEPALLATA* (*SEP*) subfamily genes encode proteins that interact with one another to form a complex regulatory network, that controls the development of the floral organ (e.g., sepals, petals, stamens, carpels, and ovules)^2^. Moreover, accumulating evidence suggests that the type II genes play crucial regulatory roles in various developmental processes throughout the *A*. *thaliana* life cycle, including embryogenesis^16^, flowering time^17^, pollen maturation and tube growth^18^, and seed dormancy^19^. The functions of the MADS-box family genes have been summarized in detail^20^. Although the diverse functions of MADS-box genes in annuals have been described, their roles in perennials remain relatively unknown.

Perennials survive periods of seasonally-induced stress as dormant buds^21^. However, the molecular mechanism underlying this dormancy has not been fully characterized^22^. *SHORT VEGETATIVE PHASE* (*SVP*) is a MADS-box gene that represses flowering in *A*. *thaliana*^23^. Homologs of *SVP* are known as *dormancy*-*associated MADS*-*box* (*DAM*) genes and have been identified in peach (*Prunus persica*)^24^, leafy spurge (*Euphorbia esula*)^25^, Japanese apricot (*Prunus mume*)^26^, and Japanese pear (*Pyrus pyrifolia*)^27^. For example, the *P*. *persica DAM5* and *DAM6* expression patterns correspond with dormancy induction and release. Specifically, *DAM5* and *DAM6* expression levels are up-regulated in lateral floral buds throughout autumn and are down-regulated during the dormancy release stage of the endodormancy cycle^28^. Meanwhile, the *FLOWERING LOCUS T* (*FT*), which affects flower development^29^, influences the development of dormancy^30,31^. Overexpression of poplar *FT1* in the plum (*Prunus domestica*) leads to continuous flowering as well as impaired initiation of dormancy^32^. Chromatin immunoprecipitation assays in the leafy spurge using endodormant crown buds indicated that a DAM-like protein likely binds to the CArG-boxes in the *FT* promoter regions to suppress *FT* expression as part of dormancy regulation^33^. A previous study revealed that the overexpression of mulberry *FT* induces early flowering in *A*. *thaliana*^34^, but the involvement of *MnFT* in dormancy remains unexplored.

Dormancy release requires a period of accumulative chilling, which is analogous to the chilling requirement of vernalization for flowering in winter annuals^35^. In *A*. *thaliana*, the *FLOWERING LOCUS C* (*FLC*) MADS-box gene has been extensively studied. This gene encodes a flowering repressor involved in the vernalization response^36^. The FLC protein binds to the CArG-box element in the first intron of *FT* to suppress the expression of *FT*^37^. Additionally, FLC can form a dimer with SVP to repress the floral transition in *A*. *thaliana*^38^. The *MADS AFFECTING FLOWERING 1* (*MAF1*) –*MAF5* genes are closely related to *FLC*, with *MAF1*–*MAF4* encoding floral repressors and *MAF5* encoding a floral activator in *A*. *thaliana*^39^. A recent meta-analysis of microarray data suggested that the *MAF3-*like gene expression in the underground adventitious buds of leafy spurge might be useful as an endodormancy marker^40^. An analysis of the transcription profile of apple (*Malusx domestica*) revealed that the expression of two *MdFLC-*like genes, *MdMADS135* and *MdMADS136*, increased during the winter dormancy period and decreased considerably at dormancy release^41^. Similarly, *PtFLC* expression levels in trifoliate orange (*Poncirus trifoliata*) increased after September, peaked in November, and then decreased during spring^42^. Several reports have suggested that dormancy in woody plants and vernalization in annual herbaceous plants are regulated by overlapping pathways^43–46^. Consequently, considering the similar expression profiles of *DAM* and *FLC-*like genes during the dormancy period, *FLC-*like genes are likely important for dormancy. However, there is a lack of published research regarding the roles of *FLC-*like genes related to dormancy regulation in perennials.

Mulberry (order: Rosales; family: Moraceae) is a perennial woody flowering plant that is widely cultivated in Asia, Europe, Africa, and North and South America^47^. Although mulberry is mainly used for raising silkworms, its fruit is converted into wine, juice, jam, and canned food and is also used in medicinal products^48^. Mulberry belongs to the “indirect flowering” group, in which the differentiation of flower buds occurs before dormancy, while blooming and fruit development take place after dormancy release^49^. In a previous study, 36 members of the *Morus alba* MADS-box genes were detected in the floral buds^50^. However, a comprehensive analysis of mulberry MADS-box genes based on genome sequences have not yet been reported.

The importance of MADS-box proteins for plant development prompted us to investigate their functions in mulberry plants. In the present study, we conducted a comprehensive analysis of the MADS-box gene family in the *M*. *notabilis* genome^51^. We identified 54 *MnMADS* genes, including three *FLC-*like genes, *MnMADS33*, *MnMADS50*, and *MnMADS7*. We also examined *MnMADS* phylogenetic relationships, gene structures, and encoded conserved protein motifs. The expression of type I genes was nearly undetectable, while type II genes were expressed at high levels in flower-related organs. Of these genes, *MnMADS1*, *MnMADS19*, *MnMADS33*, *MnMADS45*, and *MnMADS46* were abundantly expressed in the buds of differentiating inflorescence primordia, dormant buds, and catkins. Moreover, subcellular localization of MnFLC-like proteins was investigated by ectopic expression in tobacco cells. MnFLC-like-GFP proteins were mainly localized to the nucleus, while MnMADS33-GFP was also detected in the epicyte and epidermal cell organelles of tobacco. The ectopic and constitutive expression of *MnFLC-*like genes in *A*. *thaliana* plants resulted in late-bolting phenotypes with down-regulated expression of *FT* and *SUPPRESSOR OF OVEREXPRESSION OF CONSTANS1* (*SOC1*). Analyses of mulberry floral buds indicated that *MnMADS33* and *MnFT* expression levels increased and decreased, respectively, in cold-treated mulberry floral buds under field and artificially controlled conditions. Dormancy release was associated with the down- and up-regulated expression of *MnMADS33* and *MnFT*, respectively. MnMADS33 could directly or indirectly suppress the promoter activity of *MnFT* to repress the expression of downstream report gene *in vivo*. These results along with the conserved function of *FLC* down-regulating *FT* in other systems^37,52^, suggesting that *MnMADS33* might down-regulate the expression of *MnFT* to repress the dormancy release and flowering in mulberry. The mulberry genes described herein warrant further study to characterize their regulatory effects on flowering.

## Results

### Identification of MADS-box genes from the mulberry genome

We detected 63 *M*. *notabilis* genes based on HMM and BLASTP analyses. Nine sequences (i.e., Morus001839, Morus002012, Morus002795, Morus016120, Morus016684, Morus018169, Morus020771, Morus023144, and Morus025559) did not contain a complete MADS domain-coding sequence and were excluded from further analyses. The remaining 54 genes were considered putative *MnMADS* genes (i.e., *MnMADS1–MnMADS54*). The full-length cDNA sequences of the truncated genes (*MnMADS7*, *MnMADS16*, *MnMADS21*, *MnMADS50*, *MnMADS51*, and *MnMADS52*) were verified manually based on previously reported *de novo* transcription data^50^. The *MnMADS7*, *MnMADS16*, *MnMADS21*, *MnMADS50*, *MnMADS51*, and *MnMADS52* genes corresponded to *c79593_g1*, *c68277_g1*, *c70333_g2*, *c78979_g1*, *c79142_g3*, and *c74310_g1*, respectively. Details regarding the putative MnMADS proteins, including length (i.e., 79–509 amino acids), molecular weight, and isoelectric point, are provided in Table S1. The 54 *MnMADS* genes were detected on 42 scaffolds. Scaffolds 1731 and 336 contained the most *MnMADS* genes (three genes), followed by scaffolds 1100, 261, 262, 271, 553, 651, 73, and 948 (two genes). The other 31 scaffolds consisted of only one *MnMADS* gene. Additionally, *MnMADS* genes on the same scaffold were located relatively close to each other and apparently formed clusters.

We identified potential paralogous pairs among the 54 *MnMADS* genes using MCScanX. The *MnMADS* gene types are presented in Table S1. Eight paralogous pairs were detected, including three whole genome/segmental duplications (i.e., *MnMADS16*/*MnMADS46*, *MnMADS45*/*MnMADS53*, and *MnMADS17*/*MnMADS45*) and five tandem duplications (i.e., *MnMADS42*/*MnMADS43*, *MnMADS27*/*MnMADS28*, *MnMADS9*/*MnMADS10*, *MnMADS26*/*MnMADS27*, and *MnMADS2*/*MnMADS3*) (Table S2). The Ka/Ks ratio was calculated for the coding sequences of eight paralogous pairs to determine the type of Darwinian selection underlying the gene divergences after duplication events. The Ka/Ks ratios of whole genome/segmental duplications were <0.1, while the Ka/Ks ratios for tandem duplications were 0.03–1.57 (average: 0.58). There were two pairs of *MnMADS* genes (i.e., *MnMADS26*/*MnMADS27* and *MnMADS2*/*MnMADS3*) for which the Ka/Ks ratio was >1.

### Phylogenetic analyses of the *Arabidopsis thaliana* and mulberry MADS-box genes

**Figure 1:**
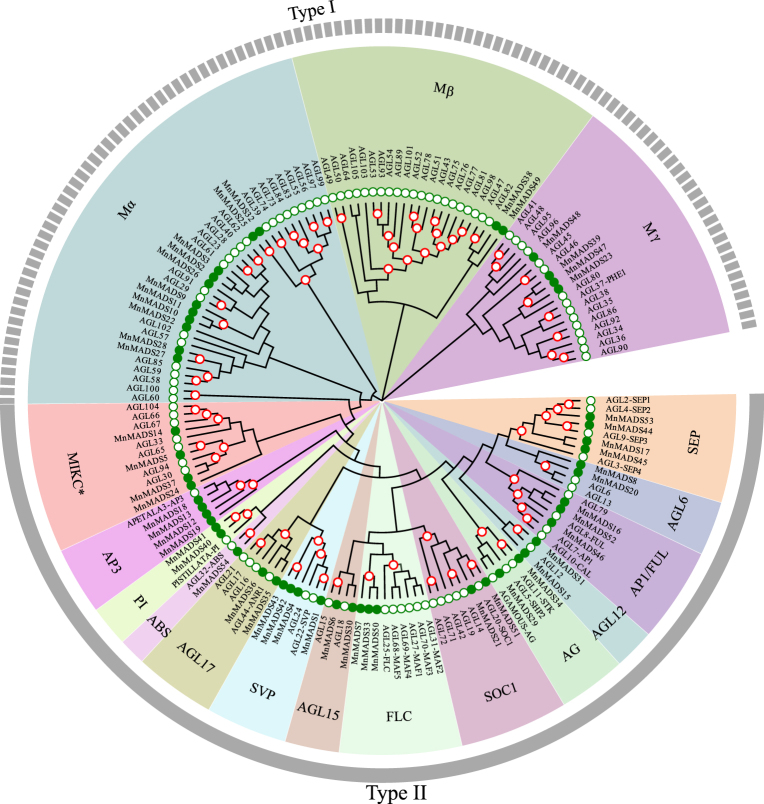
Unrooted neighbor-joining tree constructed using all full-length MADS-box proteins from mulberry (green dot) and *Arabidopsis thaliana* (green circle). Bootstrap values over 90% are labeled with red circles. The MADS-box genes are divided into two groups (i.e., type I and type II). Fourteen groups in type II are highlighted in different colors.

**Figure 2:**
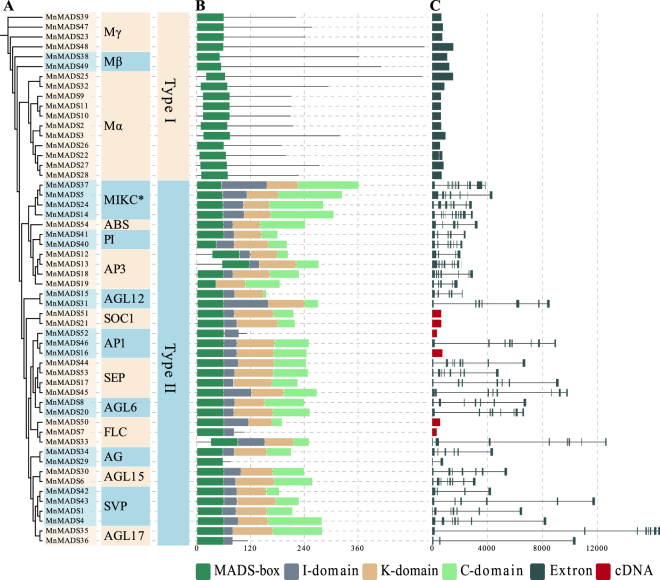
Phylogenetic relationships and structural analyses of mulberry MADS-box genes. (**A**) The unrooted maximum likelihood tree of 54 MnMADS proteins was reconstructed using a JTT + I + G model. Groups are shown in different colors. (**B**) Domains of MADS-box, intervening (I), keratin-like (K), and C-terminal (C) are indicated by different colored broad lines. The length corresponds to motif length. (**C**) The broad grey lines and thin lines indicate exons and introns, respectively. Broad red lines indicate the full-length cDNA of *MnMADS* genes, which are manually modified according to the *de novo* transcriptome assembly data reported by Shang *et al*.^50^.

To analyze the phylogenetic relationships among *MnMADS* genes, an unrooted neighbor-joining (NJ) tree was constructed based on the alignment of the full-length amino acid sequences of 54 putative MnMADS proteins and 103 *A*. *thaliana* MADS-box transcription factors. A phylogenetic analysis revealed that 17 type I and 37 type II MADS-box genes encoded the 54 MnMADS proteins (Fig. 1). According to the *A*. *thaliana* MADS-box subgroup classifications, the type II proteins were further divided into the following 14 groups: SEP, AGL6, AP1/FUL, AGL12, AG, SOC1, FLC, AGL15, SVP, AGL17, ARABIDOPSIS BSISTER (ABS), PI, AP3, and MIKC*. Most of these subgroups were named according to extensively investigated MADS-box genes. To further validate the evolutionary relationships among MnMADSs, an unrooted NJ tree was constructed using the MADS-box protein sequences of *A*. *thaliana*, grape, and mulberry (Supplementary Fig. S1). Moreover, the evolutionary relationships among the 54 MnMADS proteins were investigated using a maximum likelihood (ML) method (Fig. 2A). It is noteworthy that the FLC subfamily was adjacent to AP1, SEP, and AGL6 in the ML tree. Furthermore, the evolutionary relationships of the MnMADS proteins in Fig. 2A were consistent with the relationships in the NJ phylogenetic tree (Fig. 1).

### Mulberry MADS-box protein domains, gene structures, and classes

All MnMADS proteins consisted of a MADS-box domain. As shown in Fig. 2B, except for MnMADS7, MnMADS29, MnMADS36, and MnMADS52, all the type II MnMADS proteins contained a K-domain. Additionally, the *MnMADS* gene structures were investigated using comparative alignments of the corresponding genomic and coding sequences. The number of introns varied from 0 to 10, with genes in the same family containing a similar number of introns. Most of the type I genes had no introns, while the majority of type II genes had six. The genes indicated by broad red lines in Fig. 2C are manually corrected cDNA sequences. For most MADS-box gene families and subfamilies, there were more *A*. *thaliana* genes than mulberry genes (or an equal number). For example, we identified 17 type I mulberry genes (31% of the total number of *MnMADS* genes), while there were 58 type I *A*. *thaliana* genes (56% of the total number of *A*. *thaliana* MADS-box genes) (Supplementary Fig. S2). In contrast, the *AP3* and *PI* subfamilies contained more mulberry genes than *A*. *thaliana* genes. We observed that *A*. *thaliana* carried only one *AP3* and one *PI* gene, while mulberry contained four *AP3* and two *PI* genes. Additionally, mulberry also had four *SVP/AGL24* genes, while *A*. *thaliana* consisted of only two.

### Expression profiles of mulberry MADS-box genes in various organs and flower-related tissues

**Figure 3:**
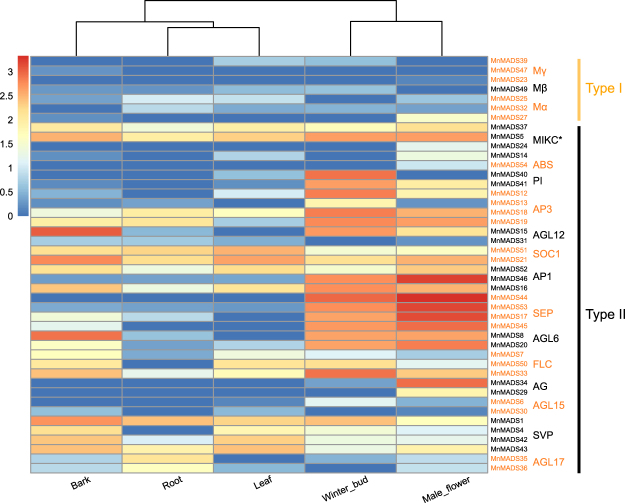
Expression profiles of *MnMADS* genes in different organs. Mulberry roots, bark, winter buds, male flowers, and leaves were used. The homologs of the corresponding mulberry genes were separately presented with black and orange colors. The names of subfamily were marked on the right side. The color scale bar at the bottom left of the figure represents log2 (RPKM+1) values.

Analyses of the *MnMADS* expression profiles in the roots, leaves, bark, winter buds, and male flowers resulted in the identification of 44 *MnMADS* genes that were expressed in at least one of these organs, including all 37 type II genes. The expression level of seven type I genes was generally low, while the expression of the remaining 10 type I genes was undetectable in all examined organs. Overall, the *MnMADS* expression levels were lowest in the roots, followed by the leaves. Most genes with more than 50 RPKM were associated with the ABCDE flowering model and were preferentially expressed in winter buds and male flowers. Several genes exhibited high expression levels in various tissues. The most highly expressed gene was *MnMADS44*, with an expression level of 535 RPKM in male flowers. It was also highly expressed in winter buds. *MnMADS46* was the next most highly expressed gene, with an expression level of 304 RPKM in male flowers. The most highly expressed gene in bark was *MnMADS15* (147 RPKM). Additionally, the expression level of *MnMADS33* (i.e., *FLC* homolog) was biased in winter buds (>80 RPKM), while the expression level of the *SVP* subfamily *MnMADS* genes was relatively low (Fig. 3, Table S3).

**Figure 4:**
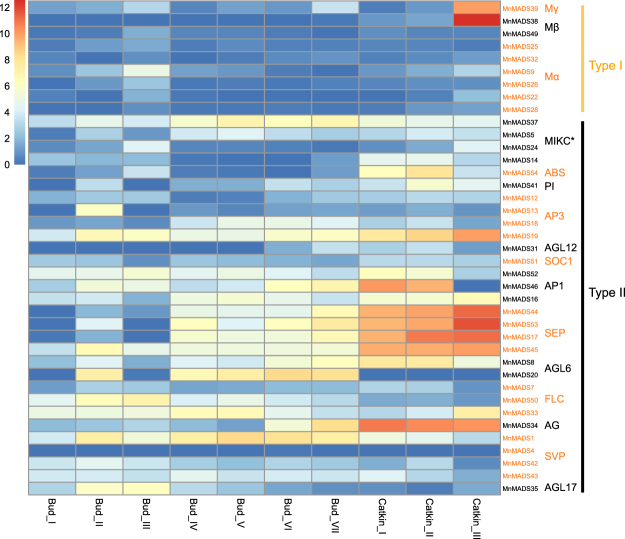
The expression of *MnMADS* genes in the flower-related tissues during inflorescence development. Bud I, II, and III represent initial, mid-term, and later stage of differentiating inflorescence primordial, respectively. Paraffin sections were prepared to ensure the correct stages (Supplementary Fig. S7). Bud IV and V are dormant buds. Bud VI and VII were dormancy release buds. Catkin I, catkin II, and catkin III were before, mid-term, and later stage of pollination, respectively. The data presented here represent two biological replicate experiments. The qRT-PCR is repeated three times for each biological replicate. *RPL15* is used as the internal control. This heat map is created by method mentioned in Fig. 3.

To further associate *MnMADS* functions with specific developmental processes, a quantitative real-time polymerase chain reaction (qRT-PCR) was used to analyze expression profiles in various flower-related mulberry tissues. Of the 54 *MnMADS* genes, 39 were expressed in at least one tissue of the mulberry species ‘Jinqiang 63’ (JQ63) (Table S4). Gene expression patterns for the buds of differentiating inflorescence primordia (buds I-III), during endodormancy and dormancy release (buds IV-VII), as well as in the different catkin development stages (catkins I-III) are shown in Fig. 4. Prominent among the nine genes in type I, both of *MnMADS38* and *MnMADS39* were highly expressed in catkin III. Of the type II genes, five (i.e., *MnMADS1*, 19, 33, 45, and 46) were abundantly expressed in all three stages. For example, *MnMADS33* was most highly expressed in catkin III but also expressed in buds I–III and buds IV-V. Meanwhile, *MnMADS1* (i.e., *SVP* homolog) was abundantly expressed in bud II and III, as well as in catkin I. The mulberry genome includes four *MnMADS* homologs of *SVP*/*AGL2*4 (i.e., *MnMADS1*, *4*, 42, and 43). We observed that *MnMADS1* was expressed at a continuously high level during the endodormancy and dormancy release periods. The remaining genes in the *SVP/GAL24* family were expressed at very low expression levels during these periods.

### *FLOWERING LOCUS C* homologs in mulberry

**Figure 5:**
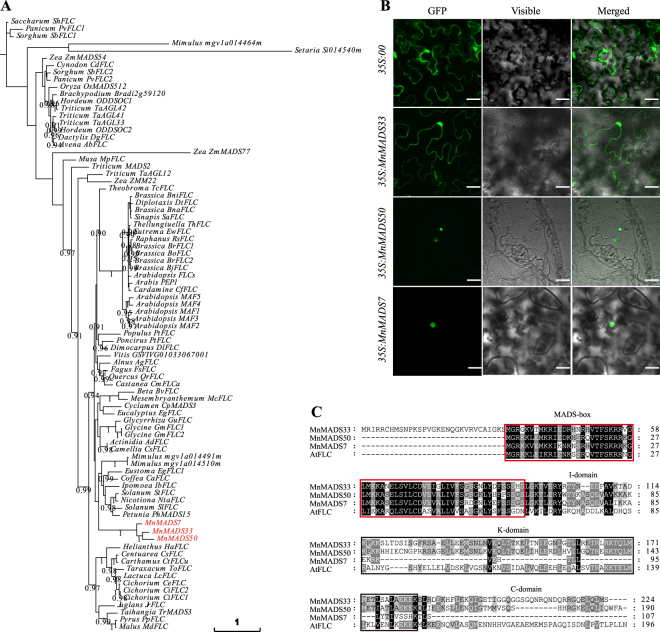
Mulberry *FLOWERING LOCUS C-like* genes. (**A**) Phylogenetic relationship of FLC homologs from multi-species. Phylogenetic tree of FLC homologs is generated using PhyML (version 3.1) by maximum likelihood method with GTR + I + G model. The mulberry genes are marked in red. (**B**) Analyses of subcellular localization of MnMADS33, MnMADS50, and MnMADS7 in *Nicotiana benthamiana* leaves. *Agrobacterium*-infiltrated tobacco leaves, expressing the MnMADS33-GFP, MnMADS50-GFP, and MnMADS7-GFP fusion proteins driven by the CaMV 35S promoter, were used for obtaining images under green fluorescence, merged light, and visible light. *35S:00* represent the empty expression vector. *Scale bar* = 25 μm. (**C**) Comparison of *A*. *thaliana* FLC, MnMADS33, MnMADS50, and MnMADS7 amino acid sequences. The MADS-box, I, K, and C-terminal domains are labelled.

**Figure 6:**
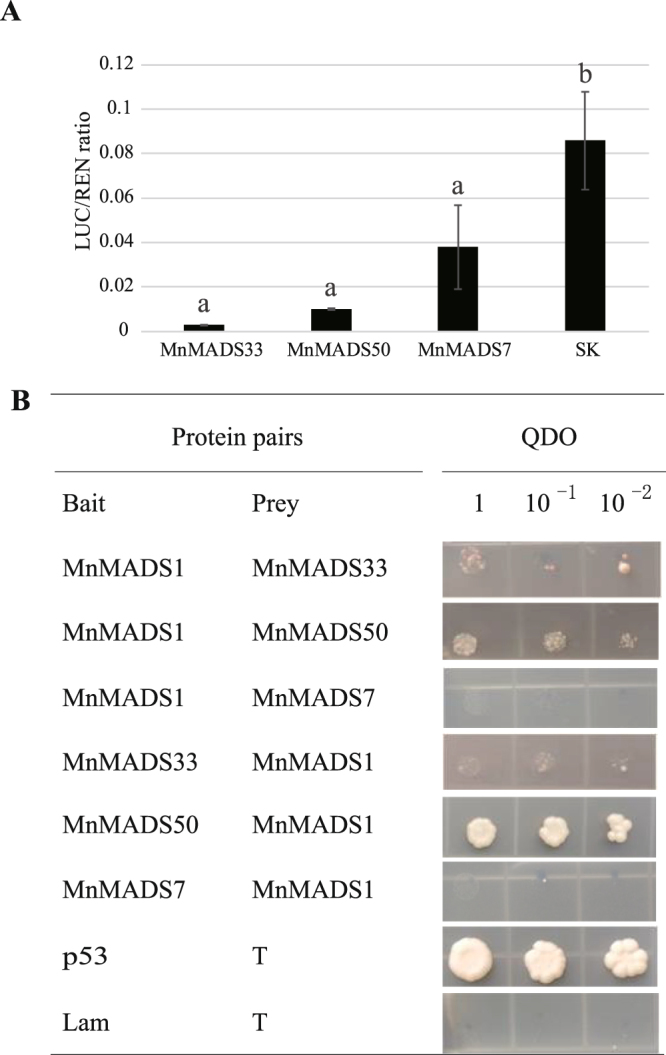
*In vivo* interaction of MnFLC-like proteins with *MnFT* promoter and MnMADS1 protein. (**A**) *In vivo* associations of MnFLC-like proteins and MnFT promoter obtained from transient assays in tobacco leaves using dual luciferase assay. SK represents empty vector. Error bars indicate SE from three biological replicates (*P* < 0.05). (**B**) Yeast co-transformation interaction assessment between MnFLC-like and SVP-like (MnMADS1). Yeasts were plated on quadruple dropout (QDO) medium after diluting 1, 10, and 100 times.

To provide additional evidence for the relationship between FLC and MnMADS33/MnMADS50/MnMADS7, we reanalyzed the previously reported phylogenetic relationships among the FLC-like proteins from mulberry and other species^53^. MnMADS33, MnMADS50, and MnMADS7 were clustered in the same clade and were closely related to the *Mimulus guttatus* FLC homologs (Fig. 5A). An investigation of the subcellular localization in *N*. *benthamiana* leaves indicated that MnMADS50 and MnMADS7 were mainly located in the nucleus. Meanwhile, MnMADS33 localized to the epicyte and organelles including guard cells, but primarily the nucleus (Fig. 5B). Analyses of protein structures revealed that MnMADS33 and MnMADS50 contained the classic MIKC motifs. Additionally, MnMADS7 comprised the MADS-box and I- domains, but the K- and C- domains were incomplete (Figs 2 and 5C). An amino acid sequence alignment confirmed that there are 31 amino acids before the MnMADS33 MADS-box domain. Moreover, we observed a 51.08% amino acid identity between MnMADS33 and MnMADS50. In contrast, *A*. *thaliana* FLC shared only 29.00% and 37.00% amino acid sequence identity with MnMADS33 and MnMADS50, respectively (Fig. 5C). Dual luciferase assays indicated that MnFLC-like proteins (i.e., MnMADS33, 50, and 7) could directly or indirectly suppress the promoter activity of MnFT and repressed the expression of the *luciferase* gene (Fig. 6A). According to the yeast two-hybrid assays, the interaction between MnMADS50 and MnMADS1 is strong. In addition, a very weak interaction between MnMADS33 and MnMADS1, and no interaction between MnMADS7 and MnMADS1 were detected (Fig. 6B). β-galactosidase activity assays were further conducted to confirm the true interactions between MnMADS33 and MnMADS50 with MnMADS1. The β-galactosidase activity for the interaction combinations pGBKT7-MnMADS1/pGADT7-MnMADS33, pGBKT7-MnMADS1/pGADT7-MnMADS50, pGADT7-MnMADS1/pGBKT7-MnMADS33, pGADT7-MnMADS1/pGBKT7-MnMADS50, pGADT7-T7/pGBKT7-p53, and pGADT7-T7/pGBKT7-lam were 17.51, 15.30, 15.06, 16.27, 15.68, and 1.00 units, respectively (Supplementary Fig. S3).

### Ectopic expression of mulberry *FLOWERING LOCUS C*-like genes in *Arabidopsis thaliana* resulted in a late-bolting phenotype

**Figure 7:**
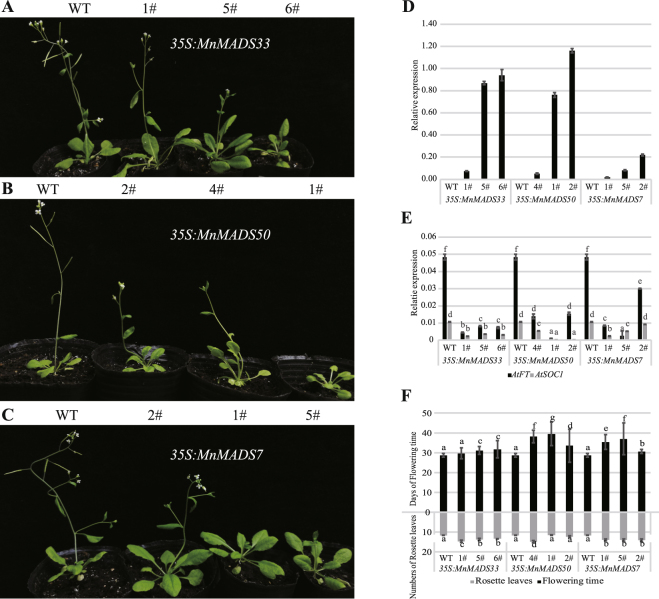
Phenotypes of the wild-type and transgenic *Arabidopsis thaliana* plants overexpressing *MnMADS33*, *MnMADS50*, and *MnMADS7*. (**A**–**C**) Five-week-old wild and transgenic seedlings under long-day conditions (16/8 h of light/dark). (**D**) Expression levels of *MnMADS33*, *MnMADS50*, and *MnMADS7* determined by qRT-PCR in wild-type and transgenic *A*. *thaliana*. (**E**) qRT-PCR analyses of *AtSOC1* and *AtFT* in wild-type and transgenic *A*. *thaliana*. (**F**) The leaf numbers of rosette in wild and transgenic *A*. *thaliana* plants. For qRT-PCR analyses in (**D**,**E**), *A*. *thaliana Actin7* is used as the internal control. Error bars represent the standard deviations of three independent experiments. Significant differences are indicated by different lower-case letters resulting from one-way Duncan’s test (P < 0.05).

To investigate the potential functions of *MnMADS33*, *MnMADS50*, and *MnMADS7* genes regarding the flowering transition, transgenic *A*. *thaliana* plants expressing these genes under the control of the CaMV 35S promoter were examined. We selected three transgenic lines for each gene and analyzed their flowering phenotypes and gene expression levels (Fig. 7A–C). Gene-specific primers were used to conduct qRT-PCR analyses of WT and transgenic *A*. *thaliana* to validate the phenotypic assessment of the transgenic lines and the expression of flowering-related genes (Fig. 7D,E). The *FT* and *SOC1* expression levels were lower in all transgenic lines than in the WT (Fig. 7E). Meanwhile, the transgenic lines exhibited varying degrees of delayed flowering and produced more rosette leaves than those of the the WT plants (Fig. 7F).

### Relationships between endodormancy and the *FLOWERING LOCUS C*- and *FT*-like genes in mulberry

**Figure 8:**
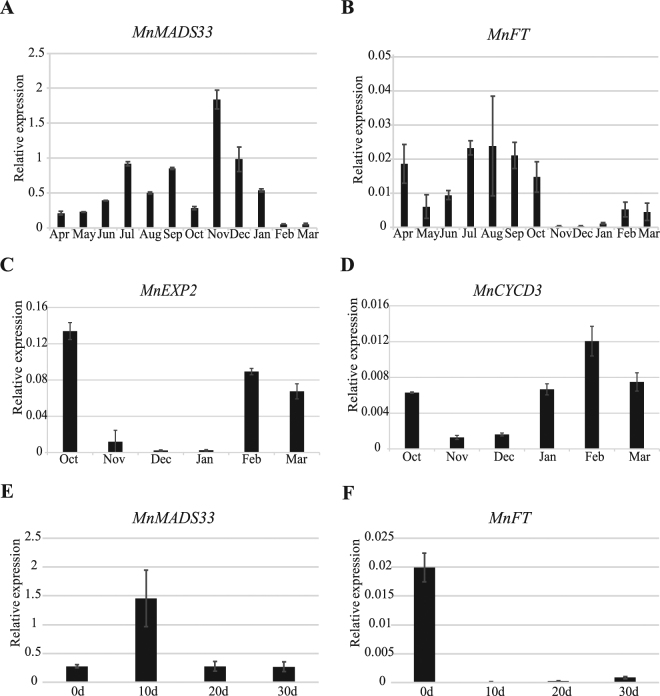
Expression of *MnMADS33* and *MnFT* genes in floral buds. (**A**,**B**) Expression of *MnMADS33* and *MnFT* in adult mulberry buds over one year. (**C**,**D**) Expression analyses of *MnEXP2* and *MnCYCD3* in the mulberry floral buds during endodormancy. (**E**,**F**) Expression of *MnMADS33* and *MnFT* in mulberry floral buds under controlled cold conditions in 30 days. Gene expression is measured by qRT-PCR using the *RPL15* as a reference (n = 3, mean ± measurement range).

**Figure 9:**
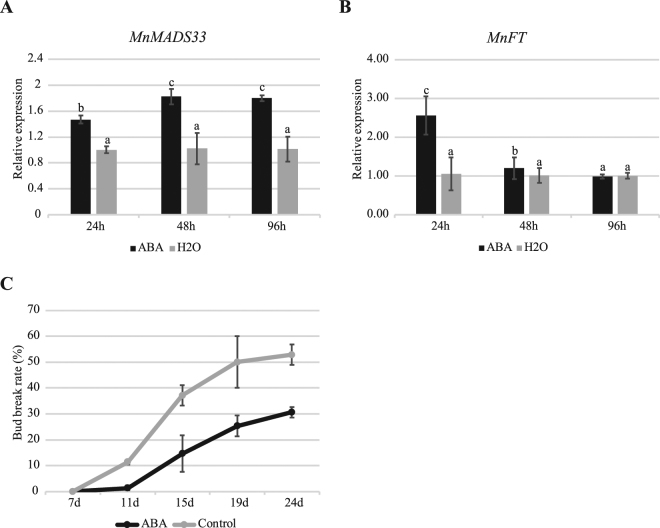
Abscisic acid treatment influences the expression of *MnMADS33* and *MnFT*. (**A**,**B**) Expression profiles of *MnMADS33* and *MnFT* after treatment for 24, 48, and 96 hours. (**C**) The break rate of dormant buds after treatment for 7, 11, 15, 19, and 24 days. Gene expression is measured by method mentioned in Fig. 8.

A qRT-PCR was conducted to clarify the relationship between the seasonal periodicity of floral bud development and the expression of *MnFLC*-like genes and *MnFT*-like gene (Fig. 8A,B, Supplementary Fig. S4). An expansion gene (*MnEXP2*; Morus011237) and a cyclin D-type gene (*MnCYCD3*; Morus027452) were used to provide evidence of the endodormancy cycle activities^54^. The *MnEXP2* and *MnCYCD3* expression levels were rapidly down-regulated in November and recovered during dormancy release (Fig. 8C,D). Meanwhile, the *MnMADS33* and *MnFT* expression levels fluctuated before endodormancy, after which *MnMADS33* expression was sharply up-regulated, peaking in November, and then down-regulated until dormancy release in the spring. The *MnFT* gene exhibited a contrasting expression pattern during the same period. The expression of *MnMADS50* and *MnMADS7* were relatively low during endodormancy (Supplementary Fig. S4). An analysis of floral buds of plants grown under artificially controlled cold conditions revealed that *MnMADS33* expression was rapidly up-regulated after 10 days of chilling, while *MnFT* exhibited the opposite expression pattern (Fig. 8E,F). We also investigated the expression levels of genes related to ABA biosynthesis and catabolism [e.g., ABA 8-hydroxylase gene (*MnCYP707A4*; Morus027528), RING-H2-type zinc-finger gene (*MnXERICO*; Morus006867), and 9-cis-epoxycarotenoid dioxygenase gene (*MnNCED1*; Morus012507)] in mulberry floral buds exposed to low temperatures under field and artificially controlled conditions. The genes exhibited similar expression profiles under the two experimental conditions (Supplementary Fig. S5). The *MnCYP707A4* and *MnNCED1* expression levels were up-regulated after endodormancy, and then down-regulated before dormancy release. The expression of *MnNCED1* coincided with that of *MnMADS33*. Furthermore, *MnXERICO* expression was down-regulated during endodormancy and up-regulated before dormancy release. Mulberry floral buds were treated with ABA to confirm the relationships between ABA and the expression of *MnMADS33* and *MnFT* (Fig. 9). The ABA treatment up-regulated the expression of *MnMADS33* and *MnFT*; however, while the increased *MnMADS33* expression level was stable, the ABA-induced *MnFT* expression level gradually decreased (Fig. 9A,B). The expression of *MnMADS50* and *MnMADS7* was up-regulated at 24 h and then down-regulated at 48 h and 96 h after ABA treatment (Supplementary Fig. S6). Furthermore, bud break was repressed by ABA treatment (Fig. 9C).

## Discussion

Woody plants are characterized by a long growth cycle. Mulberry has a moderately long juvenile phase, and starts to flower 2–3 years after seeds are sown^55^. The extensive use of mulberry fruit in food and medicinal products compelled us to focus on mulberry flower development, which is partly influenced by the MADS-box gene family. Advances in whole-genome sequencing technology have facilitated the comprehensive analysis of this particular gene family. In the present study, we used systematic bioinformatics analyses and molecular methods to investigate the role of the mulberry MADS-box gene family in terms of floral transition and flower bud development.

### Mulberry MADS-box family genes are conserved in structures and functions during evolution

We identified and characterized 54 *MnMADS* genes in mulberry (Table S1). The *MnMADS* gene structures and encoded protein domains were highly similar to those of the corresponding genes from other species^2,3,56–61^. The ABCDE model for annual plants is the most well-known floral development model. We detected *AP1*, *AP3*, *PI*, *AG*, *STK*, and *SEP* homologs among the identified 54 *MnMADS* genes (Figs 1 and 2, Supplementary Fig. S2). In most cases, homologs of the ABCDE model genes produced similar expression profiles (Figs 3 and 4). For example, four *SEP*-like genes in the E class were expressed in a floral-related-organ-biased manner. This result was consistent with the *SEP* function in *A*. *thaliana* floral organ development^10,13^. The *A*. *thaliana* MADS-box gene family is regulated by a complex network and is crucial for floral transitions. We detected the A-, B-, and E- class genes in the differentiating inflorescence primordia, suggesting a functional conservation among floral signal integration pathways.

### Conserved evolution of mulberry type I and type II MADS-box genes

Despite the lack of an assembled genome, 10 clusters containing 22 genes were detected based on their locations on the scaffolds (Table S1). A previous study involving synteny network analyses indicated that *AGL6-SOC1* and *SEP-AP1* tandems are conserved across all angiosperms^62^. In the present study, we observed one *AGL6-SOC1* and three *SEP-AP1* tandems. Moreover, we detected three tandem duplication clusters, including eight Mα genes, while no duplication events were observed in the Mβ and Mγ subfamilies. This may explain why there are more Mα genes than Mβ and Mγ genes in mulberry. An analysis of the evolution of *A*. *thaliana* and *Oryza sativa* MADS-box genes suggested that type I genes underwent a faster birth-and-death evolution than type II genes, in part because of a higher frequency of segmental gene duplication and weaker purifying selection in type I than in type II genes^63^. Generally, Ka/Ks = 1 indicates a neutral selection, Ka/Ks <1 corresponds to a purifying selection, and Ka/Ks >1 suggests an accelerated evolution with positive selection^64^. In our study, the Ka/Ks ratios of three whole genome/segmental duplication paralogs from type II were <0.1, consistent with a strong purifying selection pressure. The paralogous pairs of the type I Mα subfamily genes had a higher Ka/Ks ratio, including two pairs (*MnMADS26*/*MnMADS27* and *MnMADS2*/*MnMADS3*) with Ka/Ks ratios >1, suggesting the Mα genes evolved under a positive selection pressure. There were three pairs of *MIKC*^*c*^ genes distributed in a tandem duplication pattern. According to the duplication types of the paralogous pairs (Table S2), the expansion of the type I gene group was due to tandem duplications, whereas segmental duplications played a leading role in the expansion of the type II gene group. The expansion patterns of the mulberry type I and type II MADS-box genes are similar to those reported for *A*. *thaliana*^3^, *O*. *sativa*^65^, and *P*. *mume*^58^.

### Identification of mulberry *FLOWERING LOCUS C*-like genes

A previous study concluded that FLC functions as a flowering repressor, which delays flowering by inhibiting the expression of the activators of flowering such as *FT* and *SOC1*^37^. A recent transcriptome study in mulberry determined that *MnMADS33*, *MnMADS50*, and *MnMADS7* (i.e., *c75631_g1*, *c78979_g1*, and *c79593_g1*, respectively) are *AGL6* homologs based on gene annotations^50^. In the present study, *MnMADS33*, *MnMADS50*, and *MnMADS7* were identified as *FLC-*like genes according to genome-wide identification and re-analysis of the phylogenetic relationships among *MnMADS* genes (Table S1). Our data was further verified by completing a multi-species phylogenetic analysis (Fig. 5). The mulberry *FT* promoter has been cloned^66^. Four CArG-box like elements were identified in the *MnFT* promoter (Supplementary File 1). MnFLC-like proteins can directly or indirectly suppress the *MnFT* promoter activity and suppressed the expression of the *luciferase* gene in the dual luciferase assay (Fig. 6A). Moreover, the interactions of MnMADS33/MnMADS1 and MnMADS50/MnMADS1 were detected. All these results demonstrated that *MnMADS33*, *MnMADS50*, and *MnMADS7* might be *FLC*-like genes. Although, ectopically expressed *MnMADS33*, *MnMADS50*, or *MnMADS7* in *A*. *thaliana* slightly delayed flowering, the heterologous system results in a weaker phenotype than expected potentially because the binding affinity to the interacting partner or target DNA might be weaker than the original one.

### *MnMADS33* may down-regulate the expression of mulberry *FLOWERING LOCUS T* in dormant buds to inhibit dormancy release

In *A*. *thaliana*, *FT* is expressed in leaves and the resulting protein migrates to the shoot apex where it induces the formation of floral primordial^67^. In *P*. *persica*, *DAM5* and *DAM6* expression levels are up-regulated during dormancy development and down-regulated during the winter chilling period^68^. A recent study suggested that DAM5 and DAM6 are key regulators of dormancy^35^. Similarly, we observed that *MnMADS33* expression in mulberry floral buds is dramatically up-regulated after endodormancy and then down-regulated, while *MnFT* exhibits the opposite expression pattern (Fig. 8), suggesting that *MnMADS33* affects dormancy and negatively regulates the expression of *MnFT* during the dormancy period.

For plants in the indirect flowering group, such as mulberry, the formation and development of the inflorescence primordia occur in the buds in summer and then cease growth during endodormancy until next bud burst in spring. Additionally, dormant mulberry buds contain floral and leaf primordia^50^. For the relatively high *MnMADS33* and *MnMADS50* expression levels in differentiating inflorescence primordial, we propose that the encoded proteins help to maintain the inflorescence meristems in buds and inhibit bud burst and flowering by repressing the localized expression of *MnFT*. Before endodormancy, the *MnFT* and *MnMADS33* expression levels fluctuate in floral buds, and the balance between these levels likely prevents the bursting of the floral buds. *MnMADS50*, *MnMADS7*, and *MnMADS33* are three *FLC*-like genes. However, the low expression level in floral buds during endodormancy gathered with the down-regulation by ABA indicated that *MnMADS50* and *MnMADS7* had little relationship to endodormancy.

A recent study suggested that *P*. *pyrifolia PpDAM1* up-regulates *PpNCED3* expression and forms a feedback regulatory loop with ABA metabolism and signaling pathways to control endodormancy^69^. In *A*. *thaliana*, *ABSCISIC ACID-INSENSITIVE 4* (*ABI4*), which is a key component of the ABA signaling pathway, directly promotes *FLC* transcription and negatively regulates the floral transition^70^. In our study, *MnNCED1* expression was up-regulated which is consistent with the hypothesis that high *MnMADS33* expression level during the induction of endodormancy up-regulates ABA signaling and promotes endodormancy induction (Supplementary Fig. S4). Moreover, an ABA treatment up-regulated *MnMADS33* and *MnFT* expression, although the *MnFT* expression level was subsequently down-regulated at 48 and 96 h after treatment (Fig. 9). Our results support the hypothesis that dormancy and flowering share overlapping pathways^46^. We also provide evidence supporting the hypothesis that *FLC-*like genes affect dormancy by directly or indirectly down-regulating the expression of *FT-*like genes. However, whether *MnMADS33* and *MnFT* function in the same regulatory pathway or act independently during dormancy remains to be determined.

## Conclusions

In summary, 54 putative MADS-box family members were identified in the mulberry genome. We investigated the *MnMADS* phylogenetic relationships, gene structures, and encoded protein domains. A comparison of the expression patterns of paralogous pairs provided insights into the functional conservation of MADS-box proteins from mulberry and other species. Mulberry FLC-like proteins (i.e., MnMADS33, MnMADS50, and MnMADS7) were mainly localized to the nucleus, although MnMADS33 was also detected in the epicyte and organelles. MnFLC-like proteins directly or indirectly suppressed the promoter activity of the mulberry *MnFT* gene *in vivo*. Phenotypic analyses of WT and *MnFLC-*like*-*overexpressing *A*. *thaliana* plants indicated that the transgenic plants exhibited delayed flowering and down-regulated *FT* and *SOC1* expression levels. Additionally, we propose that *MnMADS33* might interact with *MnFT* to regulate dormancy and flowering. The data presented herein enhance our understanding of the roles of *MnMADS* genes related to the regulation of flowering.

## Methods

### Plant material

Mulberry JQ63 was used for gene expression analysis and vector construction. Mulberry materials were obtained from the Southwestern University mulberry germplasm for field sampling. Floral buds were collected before, during, and after the differentiation of inflorescence primordia (termed bud I, bud II, and bud III) in April 2016. Paraffin sections were prepared to ensure correct stages (Supplementary Fig. S7)^71^. Floral buds during endodormancy (November and December 2015) (as bud IV and bud V) and dormancy releasing stages (January and February 2016) (termed bud VI and bud VII) were gathered. Mulberry catkins were collected in three development stages (before, during, and after pollinated stages; termed catkin I, catkin II, and catkin III) in May 2016. Mulberry cuttings for chilling and abscisic acid (ABA) treatment were obtained on September 28, 2016 and November 4, 2016, respectively. For chilling treatment, cuttings were exposed in 4 °C and were sampled once per 10 days for total RNA extraction. For ABA treatment, cuttings were immersed in vases with 10 μM ABA (Sangon Biotech, Shanghai, China) solution in a growth chamber at 24 °C under a 14/10 h light/dark regime. After incubation for 96 h, cuttings were transferred to tap water. The control was treated with water all the time.

### Identification of mulberry MADS-box genes

We searched the *M*. *notabilis* genome database (http://morus.swu.edu.cn/morusdb/) to identify MADS-box genes. Information and sequences of *A*. *thaliana* MADS-box proteins were downloaded from The Arabidopsis Information Resource at website (https://www.arabidopsis.org/). To identify the maximum number of MADS-box domain-containing sequences, the mulberry peptide database was searched using two methods. First, all 107 *A*. *thaliana* MADS-box proteins were used in a BLASTP search (e-value of 1e−10)^72^. Second, the hidden Markov model (HMM) profile of the MADS-box domain (accession no. PF00319) was downloaded from the Pfam database (http://pfam.xfam.org/)^73^. The HMMER (version v3.1b2)^74^ was used to search MADS-box genes in the mulberry database using the HMM profile. All sequences obtained using above two methods were used as queries to BLAST the *de novo* transcriptome assembly data^50^. The truncated sequences were manually corrected based on the *de novo* transcriptome assembly data. We used the GENSCAN web server (http://genes.mit.edu/GENSCAN.html)^75^ to analyze the nucleotide sequences. The gene sequences were uploaded to the website to predict the coding regions, with *A*. *thaliana* as the reference organism. Furthermore, the protein sequences were analyzed on the SMART website (http://smart.embl-heidelberg.de/)^76^ for domain prediction. Proteins with MADS domains were considered candidate MnMADS proteins.

### Analyses of gene and protein properties

Schematic diagrams representing MADS-box gene structures (e.g., introns and exons) were generated using the Gene Structure Display Server (http://gsds.cbi.pku.edu.cn)^77^. We calculated coding sequence lengths and determined the number of amino acids, molecular weights, and isoelectric points of the encoded proteins using the Compute pI/Mw tool on the ExPASy website (http://web.expasy.org/compute_pi/)^78^. The MCScanX program^79^ was used to classify duplication types of *MnMADS* genes based on genome sequences encoding proteins and alignments with default parameters. The ratio of the number of nonsynonymous substitutions per non-synonymous site to the number of synonymous substitutions per synonymous site (Ka/Ks ratio) of each duplicated gene pair was calculated using DnaSP (version 5.10.01)^80^ on the full-length coding sequences. The STRING program (version 10.0)^81^ was used to construct possible protein–protein interaction networks based on experimental evidence with the highest confidence values (i.e., >0.900) in *A*. *thaliana* MADS-box networks. Furthermore, homologous genes encoding these interacting proteins were identified according to phylogenetic analyses. Their expression patterns in the differentiating inflorescence primordia stage were investigated by qRT-PCR.

### Phylogenetic reconstruction

Full-length protein sequences of all *A*. *thaliana*, grape, and mulberry MADS-box proteins were used to analyze their phylogenetic relationships. The amino acid sequences were aligned with ClustalW and an unrooted neighbor-joining (NJ) phylogenetic tree was constructed using a bootstrapping method (1,000 replicates) in MEGA 7.0^82^ with p-distance and complete deletion parameters. All MnMADS protein sequences were used to build maximum likelihood (ML) tree to ensure the reliability of the constructed tree. The unalignable regions at N and C terminals were manually trimmed to decrease phylogenetic noise. ProtTest 3^83^ was used to determine the best-fit model of amino acid sequence evolution, which took the JTT + I + G model under the Akaike information criterion. We used PhyML (version 3.1)^84^ to constructed ML tree with 100 bootstrap replicates. The sequence alignment analysis of *A*. *thaliana FLC*, *MnMADS33*, *MnMADS*50, and *MnMADS*7 was performed using the pairwise alignment program within the GeneDoc software^85^. Phylogenetic relationship of FLC homologs (Table S5) was generated by ML method using GTR + I + G model. The gene structures, protein structures, and all the phylogenetic trees were visualized using EvolView software on the website (http://www.evolgenius.info/evolview/)^86^.

### Gene expression analyses

We used reads per kilobase of exon per million mapped reads (RPKM) to compare the gene expression levels among samples (Table S3). Values for the roots, bark, winter floral buds, male flowers, and leaves of *MnMADS*s were obtained from RNA sequencing data (http://morus.swu.edu.cn/morusdb/), which were from single samples. A heatmap was generated based on the log2-transformed (RPKM+1) values using R project software. The expression patterns of *MnMADS*s during flowering were evaluated using qRT-PCR. Total RNA was extracted from the collected samples using TRIzol reagent (Invitrogen, Carlsbad, CA, USA). Contaminating genomic DNA in the total RNA was removed using RQ1 RNase-free DNase (Promega, Madison, WI, USA). RNA (1 μg) was used for cDNA synthesis with Moloney Murine Leukemia Virus (M-MLV) Reverse Transcriptase (Promega, Madison, WI, USA). Fourfold-diluted cDNA was used for qRT-PCR. The primers for qRT-PCR were designed with Primer Premier 5 (Table S6) and then checked by melting curves. The qRT-PCR was conducted using SYBR Premix Ex Taq II (Takara, Dalian, PR China) and the StepOnePlus Real Time PCR system (Applied Biosystems, Foster City, CA, USA), initiated by 30s at 95 °C and followed by 40 cycles of 95 °C for 5s, 60 °C for 30s, and completed with a melting-curve analysis program. The PCR mixture (20 μl total volume) comprised 10 μl of 2 × SYBR^®^ Premix Ex Taq, 0.4 μl of each primer (10 μM), 0.4 μl of 50 × ROX Reference Dye II, 2 μl of diluted cDNA and 6.8 μl PCR-grade H_2_O. The mulberry *RPL15* gene (Morus024083) was used as an internal control gene. The relative expression of genes for each sample was calculated using the formula 2^− [Ct (target gene) − Ct (control gene)]^. The log2-transformed (the relative fold changes+1) values of the 39 *MnMADS*s were visualized in heat maps prepared with R project software. The expression data of 39 *MnMADS*s were from duplicates of two biological replicates. Three biological replicates were performed for gene expression analyses in anniversary monthly floral buds, chilling-treated floral buds, and ABA-treated floral buds. The qRT-PCR analyses were performed three times for each biological replicate.

### Cellular localization

The open reading frames (ORFs) of *MnMADS33*, *MnMADS50*, and *MnMADS7* without stop codons were obtained via PCR amplification using gene-specific primers (Table S7). The purified PCR products were ligated into the pLGNL *35S*-*GFP* vector after digestion with *Kpn* I and *BamH* I enzymes. Linker sequences comprising four glycine and one serine residue with three repetitions (GGAGGAGGAGGATCAGGAGGAGGAGGATCAGGAGGAGGAGGATCA) were added between target genes and *GFP*. The fusion vectors were introduced into *Agrobacterium tumefaciens* strain GV3101 (pMP90)^87^. The *A*. *tumefaciens* cells, containing the pLGNL *35S-GFP*, pLGNL *35S-MnMADS33-GFP*, pLGNL *35S-MnMADS50-GFP*, or pLGNL *35S-MnMADS7-GFP* vectors, were grown to OD_600_ = 1.0 in LB liquid medium (10 g/L tryptone, 5 g/L yeast extract, and 10 g/L NaCl) containing 35 μg/mL rifampicin, 50 μg/mL kanamycin, and 30 ng/mL acetosyringone. Cells were collected by centrifugation at 5,000 g at room temperature for 10 min and placed for 3–5 hours at 25 °C after resuspending to OD_600_ = 0.4 using infiltration medium (2 g/L 2-morpholinoethanesulfonic acid, 1 g/L Magnesium chloride, and 30 ng/mL acetosyringone, pH 5.8). Infiltration was carried out by injecting *N*. *benthamiana* epidermal cells. The *N*. *benthamiana* samples were kept in darkness for 12 hours and then grown in a chamber at 24 °C under a 16/8 h light/dark regime for 2 days. The subcellular localization results were scanned by FV1200 laser scanning confocal microscope (Olympus, Tokyo, Japan).

### Dual luciferase assay

The promoter of the *MnFT* gene was amplified with the primers described in Table S7. Full-length MnMADS33/50/7 sequences were inserted into the pGreenII 62-SK vector (SK). The promoter of *MnFT* was inserted into the pGreenII 0800-LUC vector^88^. *N*. *benthamiana* leaves were used in dual luciferase assays. The *Agrobacterium*-mediated method was performed as mentioned in cellular localization. The firefly luciferase (LUC) and Renilla luciferase (REN) were assayed using dual luciferase assay reagents (Promega, Madison, WI, USA) after infiltration for 3 days. Three biological replicates were performed (three technical replicates in each independent experiments).

### Yeast two-hybrid and β-galactosidase activity assays

pGBKT7 (bait) and pGADT7 (prey) vectors were used for a yeast two-hybrid assay. Primers used for vector construction are listed in Table S7. The autoactivation and toxicity of both the bait and prey were tested. Positive controls were pGADT7-T and pGBKT7-53, and negative controls were pGBKT7-Lam and pGADT7-T. For each co-transformation pair, 200 ng of bait recombinant plasmid and 100 ng of prey recombinant plasmid were transformed into *Saccharomyces cerevisiae* Y2HGold, and then the cells were grown at 30 °C on synthetic medium lacking both leucine (Leu) and tryptophan (Trp) (Double Dropout, DDO). The positive clones were grown on synthetic medium without Trp, Leu, histidine (His), and adenine (Ade) (Quadruple Dropout, QDO) after diluting 1, 10, and 100 times. Three replications were conducted for each pair of proteins. The yeast strain AH109 was used for β-galactosidase activity analysis using Yeast β-galactosidase Assay Kit (Thermo Fisher Scientific, Hudson, NH, USA) following the manufacturer’s protocol. Yeast transformation and culture methods were the same as mentioned above. Three biological replicates were performed for each combination.

### Generation of transgenic *Arabidopsis thaliana* plants and expression analyses of downstream target genes

The full-length cDNA of *MnMADS33*, *MnMADS50*, and *MnMADS7* were amplified by PCR and were inserted into the *Kpn* I/*BamH* I cloning sites of a binary vector driven by the cauliflower mosaic virus (CaMV) 35S promoter and followed at the 3′ end by the nopaline synthase gene (NOS) terminator (pLGNL *35s-NOS*). The primers used for plasmid construction are listed in Table S7. Overexpression of *MnMADS33*, *MnMADS50*, and *MnMADS7* in *A*. *thaliana* was carried out in wild-type (WT) ecotype Columbia (Col-0) by *A*. *tumefaciens*-mediated plant transformation, which was performed by the floral dipping method^89^. Transformed seedlings were obtained from selective medium containing 50 mg/L kanamycin, and then transferred to soil. Homozygous lines of transgenic *A*. *thaliana* plants from T3 generation were used for further analyses. Plants were grown in chamber at 24 °C under a 16/8 h light/dark regime. Flowering time and the number of rosette leaves were recorded when the primary inflorescence was 0.5 cm long^90^. To understand the function of mulberry *FLC-*like genes in transgenic *A*. *thaliana*, the expressions of the flowering time genes, *FT* and *SOC1*, were investigated using qRT-PCR analyses. *A*. *thaliana Actin7* (At5g09810) was used as the internal control to normalize the expression levels. Conditions for total RNA isolation, reverse transcription, and qRT-PCR are the same as mentioned above. Primers used for expression analyses of downstream target genes in transgenic plants were listed in Table S6.

### Statistical analysis

Statistical analyses were performed by the SPSS (version 17.0). Three independent results were presented as mean values ± SD. Differences between samples were analyzed using one-way analysis of variance (ANOVA). Comparisons among means were made by Duncan’s test calculated at P < 0.05.

## Electronic supplementary material

Supplementary Tables and Figures
