# Supplementary material for: Identification of Morus notabilis MADS-box genes and elucidation of the roles of MnMADS33 during endodormancy
Source: Sci Rep. 2018 Apr 11;8:5860. doi: 10.1038/s41598-018-23985-0 (PMC5895635; doi:10.1038/s41598-018-23985-0)
Supplement: Supplementary file 1 — Supplementary Tables and Figures [file 41598_2018_23985_MOESM1_ESM.pdf]

Identification of *Morus notabilis* MADS-box genes and elucidation of the roles of *MnMADS33* during endodormancy

Yiwei Luo, **Hongshun Li**, **Zhonghuai Xiang**, **Ningjia He**[*]

State Key Laboratory of Silkworm Genome Biology, Southwest University, Beibei, Chongqing 400715, P. R. China.

[*]Correspondingauthor: Ningjia He

Tel: +86-23-6825-0797

Fax: +86-23-6825-1128

Email addresses: hejia@swu.edu.cn

Supplementary File 1

Promoter region of *FLOWERING LOCUS T* (*FT*) gene of *Morus alba*. GenBank accession number KU550086. The transcription start site is marked in blue. Four CArG-box-like elements, two of which are overlapping, are marked in red at the upstream of transcription start site.

> Promoter region of *M. alba FLOWERING LOCUS T* (*FT*) gene

GGTTGTGGGAAGTGCGTACTCGAATATGGCTGTTCTCCAGTGAGTGCACATCAGAAAACTA
ATGACAACACAACAACAATAAAAGACAAATTAAGTTGGTTCCAAGTCATGTACCTCTCTTC
TAGCGTACAACATACATACATACATACATACATGTGCACGGCACTAATGAGTGAACTAGCTA
GTACTTGGCATGTCAAAAATTTTTGAATCAATATTCTCTCAAAAGATTGAAATATTAATATATG
GTCTTTAATATACATTACACATTATACACGACACACAGATACATGCACACTGATTAATTTGAC
CAATATTTATGCATTGATCACAACATATATAATGAAGTCTTACTTGGAATTATTAATGCAATTT
AAGGGCCTATTTAGTTCACTATTCGAATTTTTAATTCGAGTTAATATGTGATGTTATGTGAGA
ACTTTTTATTGTAATAAAAAAATTAGATGTAACTGTTTCTAAGAGCTTTTTATTGTAATAAAA
CTTAAATTGATGAAGTAAACGGGGTCTAAGTGACTTTTCTTTTTCTTTTGGTGAATAAGTTAC
GTGACTTATATATTTCAATATTAATTCCCGTGAAAGTATCAAACTTTGAAAAGGCCTCATGGA
CCGACACCTAAGCACGAGGGGCACCCGACTTTACAGTTAGCATGAAAGAGGAAGAAGGCA
TCATCACCATTTTTTTTTTTTTATTTTCTTCCTTCGTCGAGGGAAAAGAACGAATGGAATT
TACTAAGAATCTATTGATGTGTACGGCAGCAAAAAGTGAGGATAAGGGCATGCAAAGAAGT
GAAATATATAAATAGCCCTTGGGTCTCTTGAATATGAGCCTCGATATATGTTGGATTCTAAAA
ACTTGTAGTAATATATAGAGCTGTGTTTTTGTTATCAAATTAACAATATG

Supplementary Tables and Figures

Supplementary Table S1. List of mulberry MADS-box genes.

MnMADSs	Gene ID[a]	Location on Scaffoldsa	Start Position	Stop Position[b]	Strand	CDS Length (bp)	Amino Acids Length (aa)	pI	Mw (kDa)	Gene type[c]
MnMADS1	Morus008539	scaffold103	57918	64429	-	642	213	8.68	24.21	1
MnMADS2	Morus003736	scaffold1100	59789	60439	-	651	216	7.7	24.27	3
MnMADS3	Morus003737	scaffold1100	76612	77574	-	963	320	9.51	35.06	3
MnMADS4	Morus007697	scaffold1103	39355	47620	-	840	279	6.36	31.74	1
MnMADS5	Morus006491	scaffold1147	170105	174466	-	975	324	6.02	36.41	1
MnMADS6	Morus007949	scaffold1309	165433	168569	-	777	258	6.91	29.2	1
MnMADS7	Morus008448	scaffold1584	264672	*	-	324	107	10.09	12.22	1
MnMADS8	Morus002799	scaffold1640	54554	61368	-	720	239	6.56	27.85	1
MnMADS9	Morus002949	scaffold1731	62938	63573	+	636	211	9.46	23.1	3
MnMADS10	Morus002950	scaffold1731	67138	67770	+	633	210	9.23	23.09	3
MnMADS11	Morus002953	scaffold1731	82991	83629	+	639	212	9.3	23.37	2
MnMADS12	Morus007959	scaffold18	28704	30750	+	612	203	6.55	23.69	1
MnMADS13	Morus026794	scaffold184	937088	939189	+	819	272	5.93	32.11	1
MnMADS14	Morus021528	scaffold206	202681	205612	+	918	305	5.02	34.54	1
MnMADS15	Morus010116	scaffold257	70802	73001	+	468	155	9.17	17.76	1
MnMADS16	Morus016688	scaffold261	453135	*	-	738	245	8.67	28.1	2
MnMADS17	Morus016690	scaffold261	469246	478414	-	678	225	9.28	25.78	1
MnMADS18	Morus019417	scaffold262	331346	334297	+	687	228	9.04	26	1
MnMADS19	Morus019418	scaffold262	335465	337295	+	558	185	7.85	21.17	0
MnMADS20	Morus020769	scaffold271	269809	276457	+	759	252	9.26	28.63	1

MnMADS21	Morus020772	scaffold271	303424	*	-	660	219	9.3	25.22	1
MnMADS22	Morus002481	scaffold2802	71078	71709	+	600	199	6.98	21.98	1
MnMADS23	Morus022655	scaffold283	240922	241653	-	732	243	9.58	27.93	1
MnMADS24	Morus010247	scaffold286	41905	44776	-	849	282	6.21	32.12	1
MnMADS25	Morus016449	scaffold315	375633	377147	+	1515	504	4.86	54.99	1
MnMADS26	Morus021710	scaffold336	738390	738959	-	570	189	9.62	20.59	3
MnMADS27	Morus021711	scaffold336	745933	746757	-	825	274	5.25	30.12	3
MnMADS28	Morus021712	scaffold336	748866	749552	-	687	228	6.93	25.68	3
MnMADS29	Morus002013	scaffold3543	53894	54684	-	228	75	9.93	8.5	1
MnMADS30	Morus003409	scaffold391	67919	73344	-	720	239	5.28	27.06	1
MnMADS31	Morus014296	scaffold396	111914	120429	-	816	271	9.65	30.71	1
MnMADS32	Morus027455	scaffold45	545938	546822	+	885	294	8.37	33.28	1
MnMADS33	Morus009159	scaffold452	46730	59370	+	756	251	9.51	28.03	1
MnMADS34	Morus011637	scaffold453	74236	78641	-	633	210	9.62	24	1
MnMADS35	Morus014795	scaffold456	321592	338108	+	843	280	9.25	32.09	1
MnMADS36	Morus025560	scaffold485	1091892	1102285	-	345	114	9.81	12.84	1
MnMADS37	Morus015670	scaffold503	101899	105762	+	1086	361	6.36	40.8	1
MnMADS38	Morus027089	scaffold518	389838	390926	+	1089	362	9.3	40.95	1
MnMADS39	Morus018312	scaffold521	30736	31401	-	666	221	9.89	25.31	1
MnMADS40	Morus003817	scaffold553	3512	5690	+	606	201	8.34	23.21	2
MnMADS41	Morus003822	scaffold553	30466	32878	+	543	180	9.16	20.79	2
MnMADS42	Morus011377	scaffold651	78123	82385	-	555	184	8.77	21.46	3
MnMADS43	Morus011378	scaffold651	91533	103328	-	687	228	5.16	25.29	3
MnMADS44	Morus017454	scaffold689	157053	163801	-	735	244	8.8	27.83	1
MnMADS45	Morus026460	scaffold73	440680	450503	+	807	268	8.12	30.26	1
MnMADS46	Morus026461	scaffold73	457529	466497	+	753	250	8.5	29.08	1

MnMADS47	Morus023824	scaffold75	237899	238675	+	777	258	9.55	29.05	1
MnMADS48	Morus016912	scaffold758	241600	243129	-	1530	509	5.21	55.11	1
MnMADS49	Morus010768	scaffold784	80590	81828	+	1239	412	6.28	47.96	1
MnMADS50	Morus012228	scaffold83	6460	*	-	573	190	9.78	21.95	1
MnMADS51	Morus023143	scaffold87	204156	*	+	651	216	9.66	24.91	1
MnMADS52	Morus018170	scaffold948	298731	*	-	339	112	9.74	12.91	1
MnMADS53	Morus018171	scaffold948	321413	326225	-	747	248	8.95	28.52	1
MnMADS54	Morus018510	scaffold96	361921	365205	-	729	242	6.53	27.58	1

[a] Accession numbers were from http://morus.swu.edu.cn/morusdb/.

[b] The cDNA of truncated genes, MnMADS7/16/21/50/51/52, and the stop positions on the scaffold were unknown and indicated with an asterisk.

[c] Gene types were determined using MCScanX and the 0-3 correspond to singleton, dispersed (modes other than segmental, tandem, and proximal), proximal (in nearby chromosomal regions, but not adjacent), tandem (consecutive repeat), and whole genome segmental duplications (collinear genes in collinear blocks), respectively.

Supplementary Table S2. Duplication types in the eight *MnMADS* pairs.

Paralogous pairs	Ka	Ks	Ka/Ks	Purifying selection	Duplicate genes type
MnMADS16/MnMADS46	0.08	2.94	0.03	Yes	WGD/segmental
MnMADS42/MnMADS43	0.05	0.69	0.08	Yes	Tandem
MnMADS45/MnMADS53	0.12	1.53	0.08	Yes	WGD/segmental
MnMADS17/MnMADS45	0.15	1.81	0.08	Yes	WGD/segmental
MnMADS27/MnMADS28	1.89	3.08	0.61	Yes	Tandem
MnMADS9/MnMADS10	0.02	0.02	0.88	Yes	Tandem
MnMADS26/MnMADS27	1.81	1.41	1.28	No	Tandem
MnMADS2/MnMADS3	2.78	1.77	1.57	No	Tandem

Supplementary Table S3. RNA-sequencing data for *MnMADS*s expression levels.

Gene ID	Root	Leaf	Bark	Winter bud	Male flower
MnMADS1	18.79	13.9	44.2	19.88	3.57
MnMADS4	0	4.84	10.54	2.29	1.22
MnMADS5	6.73	14.63	24.8	36.87	37.86
MnMADS6	0	0.08	0	1.41	0.31
MnMADS7	0.25	2.06	3.59	1.27	0.56
MnMADS8	0.46	0	89.92	32.4	35.1
MnMADS12	0	1.09	0.33	75.55	4.93
MnMADS13	0.2	0	0.12	5.19	0.15
MnMADS14	0	0.66	0.11	0	1.91
MnMADS15	0.35	0	147.41	43.98	9.02
MnMADS16	1.98	9.09	18.08	54.35	24.47
MnMADS17	0.73	0	2.16	34.2	233.41
MnMADS18	6.93	3.36	2.06	68.7	24.93
MnMADS19	8.53	0.54	6.35	58.77	36.64
MnMADS20	0.22	0.64	3.2	33.8	76.2
MnMADS21	11.25	33.28	59.95	10.67	26.13
MnMADS23	0	0	0	0	0.08
MnMADS24	0	0	0	0	1.49
MnMADS25	1.08	0.84	0.2	0	0.48
MnMADS27	0	0	0.12	0	2.85
MnMADS29	0	0	0	0	5.03
MnMADS30	0	0.34	0.42	0	0.08
MnMADS31	0.5	0.3	0.56	0	0.15
MnMADS32	0.74	0.27	0	0.31	0.2
MnMADS33	1.63	4.18	19.07	88.75	15.48
MnMADS34	0	0	0	0.22	106.04
MnMADS35	9.25	0	0.66	0.33	0.79
MnMADS36	3.33	0.35	0.73	0	0.87
MnMADS37	2.12	6.48	7.41	3.92	10.83
MnMADS39	0	0.55	0	0.41	0
MnMADS40	0	0.4	0	97.13	0
MnMADS41	0	0.11	0.09	38.91	6.78
MnMADS42	1.18	14.99	17.86	1.98	1.63
MnMADS43	5.26	19.26	18.77	1.8	5.53
MnMADS44	0	0	0	120.97	535.32
MnMADS45	0	0	1.5	39.78	99.17
MnMADS46	0.21	0.23	0.19	54.64	304.69
MnMADS47	0	0	0.13	0	0
MnMADS49	0.13	0.39	0.16	0.44	0
MnMADS50	0	8.84	7.95	10.31	1.42
MnMADS51	13.86	24.28	10.34	2.44	3.22

MnMADS52	1.93	11.12	9.5	2.58	16.14
MnMADS53	0.22	0.16	0.27	53.08	272.48
MnMADS54	0	0	0	0	0.99

Supplementary Table S4. Expression levels of *MnMADS*s based on qRT-PCR analyses.

	Inflorescence initiation						Dormancy and Dormancy release								Infructescence					
	Bud I		Bud II		Bud III		Bud IV		Bud V		Bud VI		Bud VII		Catkin I		Catkin II		Catkin III	
	Mean	SD	Mean	SD	Mean	SD	Mean	SD	Mean	SD	Mean	SD	Mean	SD	Mean	SD	Mean	SD	Mean	SD
MnMADS1	12.72	0.31	166.13	4.85	39.17	20.02	156.41	13.66	364.16	41.29	264.5	38.4	159.13	3.26	36.48	3.27	23.97	4.76	8.39	0.51
MnMADS4	0	0	0	0	0	0	0.06	0.03	0.05	0.01	0.16	0.05	0.09	0.04	0	0	0	0	0	0
MnMADS5	0.27	0.18	6.37	0.46	1.05	0.2	13.79	2.55	18.46	4.92	9.01	1.36	5.56	0.86	7.4	1.17	14.26	0.71	9.97	0.99
MnMADS7	1.28	0.23	6.29	0.08	4.77	0.32	1.66	0.28	1.94	0.22	3.29	0.28	3.07	0.54	6.68	0.3	5.86	0.76	0.93	0.06
MnMADS8	3.42	0.17	19.93	2.81	2.28	0.67	18.25	4.06	12.65	1.12	42.67	4.2	75.82	7.73	202.78	55.39	190.31	30.82	35.17	1.81
MnMADS9	0.74	0.07	4.47	2.49	29.32	2.55	1.39	0.31	1.08	0.54	0.26	0.06	0.08	0.06	1.01	0.18	2.26	0.3	6.85	0.23
MnMADS12	2.49	0.37	4.77	0.32	5.11	1.92	0.29	0.09	0.36	0.33	2.47	0.76	5.2	2.11	5.78	0.19	7.95	1.56	6.98	0.38
MnMADS13	0	0	65.39	5.61	0	0	1.14	0.7	0.44	0.25	1.54	0.1	1.74	0.54	1.35	0.09	2.25	0.17	1	0.08
MnMADS14	4.64	0.16	2.94	0.29	3.26	0.36	0.11	0	0.02	0.02	0.08	0.05	1.22	0.41	22.56	0.32	25.99	6.32	7.79	0.09
MnMADS16	10.27	1.37	12.65	1.49	2.49	0.37	32.45	4.67	48.45	8.33	25.69	2.54	10.63	0.87	46.54	1.66	47.2	17.61	95.92	62.89
MnMADS17	0.14	0.01	2.49	0.37	0	0	49.47	1.63	31.83	3.94	41.3	1.07	108.85	2.52	735.41	451.75	1927.84	1350.32	2160.74	881.39
MnMADS18	1.12	0.4	1.61	0.17	0.64	0.44	12.39	0.61	28.33	4.47	26.7	1.79	20.03	2.2	6.29	0.08	11.18	1.17	1.65	0.01
MnMADS19	13.73	0.9	102.67	62.16	67.47	8.4	30.68	2.61	28.09	3.4	56.96	6.27	67.02	7.81	208.77	24.23	395.17	2.16	981.49	176.92
MnMADS20	0.05	0.04	155.8	11.96	0.14	0.01	104.46	20.84	174.34	65.36	364.39	7.82	289.81	17.56	0	0	0	0	0	0
MnMADS22	0.4	0.13	0.03	0.01	2.47	0.76	0.19	0.05	0.06	0.03	0.07	0.01	0.02	0.01	0.06	0.05	0.35	0.1	3.78	0.84
MnMADS24	0.64	0.44	1.61	0.17	18.37	0.67	0.53	0.2	0.47	0.1	0.4	0.11	0.16	0.04	0.76	0.05	1.87	0.38	19.24	1.61
MnMADS25	0.21	0.06	1.28	0.23	1.87	0.71	0.27	0.1	0.56	0.2	0.36	0.07	0.36	0.14	0.51	0.01	0.49	0.02	0.28	0.14
MnMADS26	0.08	0.01	1.02	0.09	4.45	0.45	0.11	0.02	0.26	0.03	0.12	0.04	0.39	0.06	0.47	0.05	0.69	0.14	0.78	0.1
MnMADS28	0	0	0	0	0.81	0.24	0.15	0.06	0.16	0.05	0.25	0.1	0.16	0.05	0.25	0.1	1.01	0.2	1.51	0.13
MnMADS31	0	0	0	0	0	0	0	0	0.05	0.04	2.28	0.67	9.61	0.77	6.06	0.15	10.31	3.22	1.3	0.3
MnMADS32	0.44	0.12	0.03	0	0.71	0.28	0.06	0.03	0.22	0.13	0.55	0.15	0.86	0.06	0.37	0.06	0.58	0.02	1.37	0.04

MnMADS33	29.4	0.31	34.41	20.02	35.34	7.18	86.64	5.13	97.56	18.75	22.32	3.39	10	0.54	7.49	1.02	14.47	1.56	162.33	5.12
MnMADS34	3.23	0.48	4.47	2.49	6.68	0.3	3.32	0.22	1.47	0.36	44.11	0.64	328.42	8.96	1838.25	132.8	1485.84	267.02	1202.88	89.25
MnMADS35	6.98	0.38	63.24	28.83	74.77	3.91	8.56	0.69	4.57	1.3	1.64	0.11	0.7	0.05	0.71	0.04	0.28	0.14	2.08	0.24
MnMADS37	9.34	0.35	25.69	6.4	14.69	4.49	51.84	3.16	128.61	44.03	80.16	10.77	119.2	34.94	38.69	10.14	24.82	3.74	21.54	1.12
MnMADS38	0	0	0	0	0	0	0.08	0.01	0	0	0	0	0.15	0.06	1.01	0.18	1.64	0.1	6055.42	1008.86
MnMADS39	1.45	0.35	2.25	0.54	7.49	1.02	0.48	0.25	1.43	0.65	1.01	0.25	10.81	1.78	0.21	0.06	1.05	0.2	1000.11	218.05
MnMADS41	0	0	9.37	1.3	0	0	2.11	0.55	2.33	0.38	10.04	2.77	6.29	0.52	11.44	2.54	49.88	26.86	21.04	3.8
MnMADS42	12.05	1.66	20.42	3.33	11.95	12.56	5.48	1.06	3.74	0.86	6.65	1.28	5.17	0.88	2.26	0.3	8.41	1.24	0.62	0.05
MnMADS43	13.45	1.37	14.74	1.9	9.75	2.26	22.27	7.42	10.71	1.98	14.91	3.22	11.94	1.7	19.21	5.88	7.46	0.17	2.25	0.17
MnMADS44	0.05	0.04	2.93	0.17	1.05	0.2	26.65	1.88	22.22	2.87	23.96	3.24	95.27	4.57	732.89	183.32	918.13	27.68	2352.17	63.65
MnMADS45	9.97	0.99	98.18	18.13	26.01	6.32	36.3	1.84	37.6	4.06	35.92	0.85	43.94	1.56	761.26	201.41	774.66	61.17	981.49	176.92
MnMADS46	5.27	1.23	38.97	15.35	29.32	2.55	9.46	2.63	13.36	1.5	71.61	2.9	120.85	24.76	1121.94	75.16	727.1	327.61	0	0
MnMADS49	0.15	0.04	0.27	0.18	2.33	0.1	0.54	0.08	0.44	0.12	0.27	0.18	0.08	0.02	0.69	0.03	0.49	0.02	0.51	0.01
MnMADS50	21.28	2.18	88.47	32.75	131.3	35.83	15.76	0.66	26.69	2.03	13.12	0.87	7.85	0.54	2.08	0.24	2.33	0.15	1.08	0.12
MnMADS51	5.2	2.11	4.64	0.16	0.71	0.28	4.29	0.7	3.37	0.66	2.39	0.15	1.64	0.1	8.39	0.51	8.44	1.61	6	3.48
MnMADS52	22.5	0.57	25.03	21.65	45.98	1.84	20.29	1.71	29.68	3.1	18.28	2.35	9.39	0.97	77.73	2.97	47.2	17.61	6.01	0.35
MnMADS53	0.08	0.02	18.37	0.67	0.02	0	74.43	17.12	18.09	2.51	72.33	12.14	197.23	34.58	633.52	152.73	849.28	15.54	3406.82	312.26
MnMADS54	0.25	0.1	1.59	0.63	11.18	1.17	0.23	0.16	0.16	0.04	0.09	0.03	1.35	0.16	79.31	1.15	252.42	19.02	10.59	0.72

Supplementary Table S5. List of sequences used in the phylogenetic analyses.

**Species**	Gene name	Identifier
Actinidia deliciosa	AdFLC	FG406624.1
Alnus glutinosa	AgFLC	FQ352883
Arabidopsis thaliana	FLC	AY850002.1
Arabidopsis thaliana	MAF1	NM202431
Arabidopsis thaliana	MAF2	AY231441
Arabidopsis thaliana	MAF3	AY231445
Arabidopsis thaliana	MAF4	AY231450
Arabidopsis thaliana	MAF5	AY231455
Arabis alpina	PEP1	FJ755930
Avena barbata	AbFLC	GR360557
Beta vulgaris	BvFLC	DQ189210
Brachypodium distachyon	Bradi2g59120	Bradi2g59120
Brassica juncea	BjFLC	KJ489426.1
Brassica napus	BnaFLC	JX901141.1
Brassica nigra	BniFLC	KJ733745.1
Brassica oleracea	BoFLC	KF797913.1
Brassica rapa	BrFLC1	EF138603
Brassica rapa	BrFLC2	FJ936112
Camellia sinensis	CsFLC	GH454305
Cardamine flexuosa	CfFLC	KC618318.1
Carthamus tinctorius	CtFLC	EL510509
Castanea mollissima	CmFLC	GO921333
Centuarea solstitialis	CsFLC	EH776903
Cichorium intybus	CeFLC	EL354164
Cichorium intybus	CiFLC1	EH709586
Cichorium intybus	CiFLC2	EH704206
Coffea arabica	CaFLC	HQ845334
Cyclamen persicum	CpMADS3	AB600233.1
Cynodon dactylon	CdFLC	ES295415.1
Dactylis glomerata	DgFLC	HO164248
Dimocarpus longan	DlFLC	KJ480952.1
Diplotaxis tenuifolia	DtFLC	KX148480.1
Eucalyptus grandis	EgFLC	HS058521.1
Eustoma exaltatum	EgFLC1	AB565507
Eutrema wasabi	EwFLC	HM639741.1
Fagus sylvatica	FsFLC	FR606704
Glycine max	GmFLC1	AI900863
Glycine max	GmFLC2	FG995813.1
Glycyrrhiza uralensis	GuFLC	FS240134
Helianthus annuus	HaFLC	GE519513
Hordeum vulgare	ODDSOC1	HM130525

Hordeum vulgare	ODDSOC2	HM130526.1
Ipomoea batatas	IbFLC	EE880701
Juglans regia	JrFLC	KF729795.1
Lactuca serriola	LcFLC	DW120350
Malus domestica	MdFLC	EB140370
Mesembryanthemum crystallinum	McFLC	BF479182
Mimulus guttatus	mgv1a014464m	mgv1a014464m
Mimulus guttatus	mgv1a014491m	mgv1a014491m
Mimulus guttatus	mgv1a014510m	mgv1a014510m
Musa paradisiaca	MpFLC	FL658504
Nicotiona tabacum	NtaFLC	EB443157
Oryza sativa	OsMADS512	HC084629
Panicum virgatum	PvFLC1	FL799640
Panicum virgatum	PvFLC2	FL926484.1
Petunia hybrida	PhMADS15	AY370529
Poncirus trifoliata	PtFLC	EY817319.1
Populus trichocarpa	PtFLC	XM_002303748.1
Pyrus pyrifolia	PpFLC	AB524589
Quercus robur	QrFLC	FR627327.1
Raphanus sativus	RsFLC	AY273160
Saccharum hybrid	ShFLC	CA295053
Setaria italica	Si014540m	Si014540m
Sinapis alba	SaFLC	EF542803.1
Solanum lycopersicum	SlFLC	AK323974
Solanum tuberosum	StFLC	BG596731
Sorghum bicolor	SbFLC1	Sb07g026180
Sorghum bicolor	SbFLC2	Sb03g044170
Taihangia rupestris	TrMADS3	EF469601
Taraxacum officinale	ToFLC	DY804936
Thellungiella halophila	ThFLC	AY957537.1
Theobroma cacao	TcFLC	CU608380
Triticum aestivum	MADS2	DQ534490.1
Triticum aestivum	TaAGL12	AB007505.1
Triticum aestivum	TaAGL33	DQ512366.1
Triticum aestivum	TaAGL41	DQ512357
Triticum aestivum	TaAGL42	DQ512358.1
Vitis vinifera	GSVIVG01033067001	GSVIVG01033067001
Zea mays	ZMM22	GRMZM2G052045
Zea mays	ZmMADS54	GRMZM2G320549
Zea mays	ZmMADS77	GRMZM2G098986

Supplementary Table S6. Primers used for quantitative real-time PCR (qRT-PCR).

Gene Name	Forward Primer (5'-3')	Reverse Primer (5'-3')
MnMADS1	GCTGGAGTTGATTCGGATAACG	GCCCCAACTTGAGAGATGTGTC
MnMADS4	GCCTCAAGCTAAGGCAGATG	GTGGTCGCAGACTGATCTGA
MnMADS5	CGCAACTGATAGGACTGCAA	CATCAGATGCTGCTTTCCAA
MnMADS7	AGTCGCTCTCATTGTCTTCTCCG	GTGCTCAACCTTGCGTTTCTCA
MnMADS8	AGCCGTGGAAAGCTCTATGA	TTCAGTTTTGGAATCTCCCG
MnMADS9	CGCCAGCACTTCTACTAATGTTGT	ATCTTCGCTTTCTTCTTCTCCTCC
MnMADS12	AGATGATTGAGAACCGGACG	GCTCTCTGATACCGGTCGAG
MnMADS13	CTACCAGGAGCTATGCCAGC	TCCGCCATAGATTCATCCTC
MnMADS14	TCAAATGGCAGAGGAGCAGA	GGTACTCCGGGTGGTTCGTA
MnMADS16	CGTCTTCTCCACCAAAGGAA	TTGAGCTTTGCATGTTCCAG
MnMADS17	TCTTTGTGATGCTGAAGTTGCG	AGGTTGGGTAGCCTCCAGTGAA
MnMADS18	TGTACCAAAGGACCTCAGG	CTGACTGATCTGCATCCGAA
MnMADS19	TTGACACCGAAACCAAATCA	TGTATGAGCTGCCGATTCAG
MnMADS20	AGAGCTCTCTGTCCTCTGCG	CTGTGTTTCGCGCTCAATAA
MnMADS22	AGAAGGAGCGATGCGAGATGG	TTCAGCAGCGACATAAACGGC
MnMADS24	AATGCCCAAGACCAAGTACG	GCCGTTCTGCTTGAAAAGTC
MnMADS25	GGGTGGCAACTGTGCTTATT	AAACGACGAAGAAGAGCCAA
MnMADS26	GAGTCCGTCGCTGACAGGTTTCTA	GCCGCAGCATTGTTCTTCTTATTC
MnMADS28	AGGGGAAAACAAAAGATTGAGATAA	CCATAGAAGGTTGAGCAAAAGAGAA
MnMADS31	AGGAGGATTGAGAATCCGGT	TTCTGCAAAGATTGCGTTTG
MnMADS32	GAGAGTGGGAGGCGTGTAAG	ATGTCGTTTTGTGGACCCAT
MnMADS33	GGCAGCGGCAACCTCTAC	AGCACTCCGGAATCCACTAATA
MnMADS34	GATAGCTCAAGTGCAAGCTCCA	GTTCTTTGACGGAAAGCGAACT

MnMADS35	GGTGTCCGTACCAAGAAGGA	AATTGGAGATTGACGGATGC
MnMADS37	CCTCATTTGGGAGTTATTCTGGTTA	TAGCACATTCAGATTGTAAGGCACG
MnMADS38	TGGGATGTTGATGGAGAACA	TTCAGCACATTCATCGGGTA
MnMADS39	CCAGGTTCAAGAACATGCCT	TTGGAACATGATCCGAGTGA
MnMADS41	GGTTTCGCTTATTATCTACGGTCCC	TCTTTTTTGGTCATTTCAATCTCAG
MnMADS42	TCTTTGTGATGCTGAGATTGCT	ACGCCTTAGTTCAGTCGTCTTC
MnMADS43	AGGGCTTTTCAAGAAGGCTC	GCCTTTCAAGAATTTGCTGC
MnMADS44	GAAACAAAGGCTGGTGGAAGGATAC	TAAGGTGGGTTCACACTCTAAGGGG
MnMADS45	GCATACACCCACCACCAATCTCA	CTGTGCCACCTTCCACATTAACC
MnMADS46	AATCGGCAGGTAACTTTTTCCAA	TAACAGCAACTTCAGCGTCACAC
MnMADS49	CGTTTATGAGCCCATTCACACC	CTTTGAAGAAGTCGGCGATGTC
MnMADS50	AAGCGAATCGAGGACAAGCA	CTAAACTTCCGGAGCTGGGG
MnMADS51	CGGTTCTCTGTGATGCTGAA	TGCTGATCACGATCTTCTGG
MnMADS52	AACAAGATCAGCAGGCAGGT	TAATCAAACCAACATGGGCA
MnMADS53	TTGAGCGTCAACTTGAGACG	GAATATCAGCCCCTGGGAAT
MnMADS54	CAAAACCACAAGGCAAGTCACC	GGCATCACAAAGCACAGAGAGC
MnCYP707A4	CATCGGAGTTCTGTTTGCTGC	TGGTCTGTGCCCATGTTAAGG
MnXERICO	AGATGACACCGAAAGCAACCG	GTCCTCCTCGCAGCCACAC
MnNCED1	CCCGACGAAACTCCCCCC	TGGATTAGGACCCACCCGAA
MnCYCD3	AACAATAAGAGGAAGATAAACGAGGAA	GATGAGACAACGGACCAGGAAT
MnEXP2	GGGGCTTGCTACGAAATGC	CTGAAGGAGACCGGGACGAT
MnCTRL9	GGCTATGTGATTTACCGTGTT	TTGGTCCAGTATGAGTTGAGAA
AtACTIN7	CAGTGTCTGGATCGGAGGAT	TGAACAATCGATGGACCTGA
AtFT	AATTGTCAGAGGGAGAGTGGC	TAGGCATCATCACCGTTCGT
AtSOC1	GCTGAAGTTTCTCTTATCATC	CCCAATGAACAATTGCGTCTC

Supplementary Table S7. Primers used for vector construction.

Gene Name	Forward Primer (5'-3')	Reverse Primer (5'-3')
For gene clone		
MnMADS33	ATGCGAATCAGACGATGCCAC	TACTCAGAAAAGTCGCAGCAG
MnMADS50	ATGGGGAGAAAGAAGTTGGA	TCAAGCGAACCATTGAATCA
MnMADS7	ATGGGGAGAAAGAAGGTGGAG	TTATGACAATGTCTTATGGGATG
For MnFT promoter		
MnFT-pro	GGTTGTGGGAAGTGCGTACTC	ACGACAAGAGGGTCTCTATCTCTAG
For overexpression vectors		
MnMADS33	*GGTACC*ATGCGAATCAGACGATGCCAC	*GGATCC*TACTCAGAAAAGTCGCAGCAG
MnMADS50	*GGTACC*ATGGGGAGAAAGAAGTTGGA	*GGATCC*TCAAGCGAACCATTGAATCA
MnMADS7	*GGTACC*ATGGGGAGAAAGAAGGTGGAG	*GGATCC*TTATGACAATGTCTTATGGGATG
For yeast-two hybrid vectors		
MnMADS33-AD	*GAATTC*ATGCGAATCAGACGATGCCAC	*GGATCC*TACTCAGAAAAGTCGCAGCAG
MnMADS50-AD	*GAATTC*ATGGGGAGAAAGAAGTTGGA	*GGATCC*TCAAGCGAACCATTGAATCA
MnMADS7-AD	*GAATTC*ATGGGGAGAAAGAAGGTGGAG	*GGATCC*TTATGACAATGTCTTATGGGATG
MnMADS1-AD	*GAATTC*ATGGCGAGAGAGAAGATTCAG	*GGATCC*TCAGCCAGAGTATGGTAGCC
For dual luciferase assay vectors		
MnFT	*AAGCTT*GGTTGTGGGAAGTGCGTACTC	*GGATCC*ACGACAAGAGGGTCTCTATCTCTAG
MnMADS33	*GGATCC*ATGCGAATCAGACGATGCCAC	*GAATTC*TACTCAGAAAAGTCGCAGCAG
MnMADS50	*GGATCC*ATGGGGAGAAAGAAGTTGGA	*GAATTC*TCAAGCGAACCATTGAATCA
MnMADS7	*GGATCC*ATGGGGAGAAAGAAGGTGGAG	*GAATTC*TTATGACAATGTCTTATGGGATG
For subcellular vectors		
MnMADS33	*GGTACC*ATGCGAATCAGACGATGCCAC	*GGATCC*TCAGAAAAGTCGCAGCAG
MnMADS50	*GGTACC*ATGGGGAGAAAGAAGTTGGA	*GGATCC*AGCGAACCATTGAATCA

MnMADS7	*GGTACC*ATGGGGAGAAAGAAGGTGGAG	*GGATCC*TGACAATGTCTTATGGGATG

Red indicate the restriction sites.

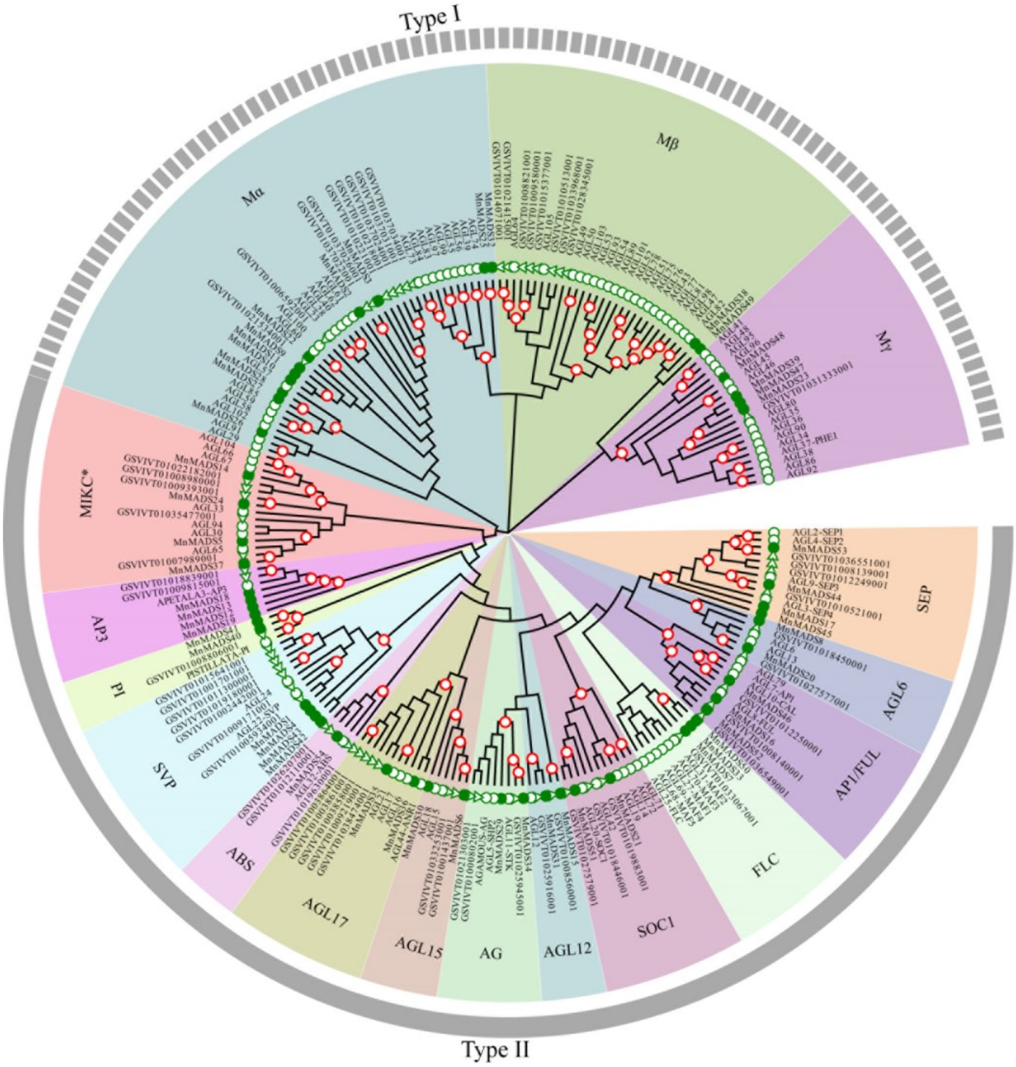

Supplementary Figure S1. Unrooted neighbor-joining tree is constructed using all full-length MADS-box proteins from mulberry (filled green circle), grape (empty green triangle), and *Arabidopsis thaliana* (empty green circle). Branches with more than 90% bootstrapping support are labeled with an empty red circle. The MADS-box genes are divided into two groups (i.e., type I and type II). The main subgroups in type II are indicated by a different background.

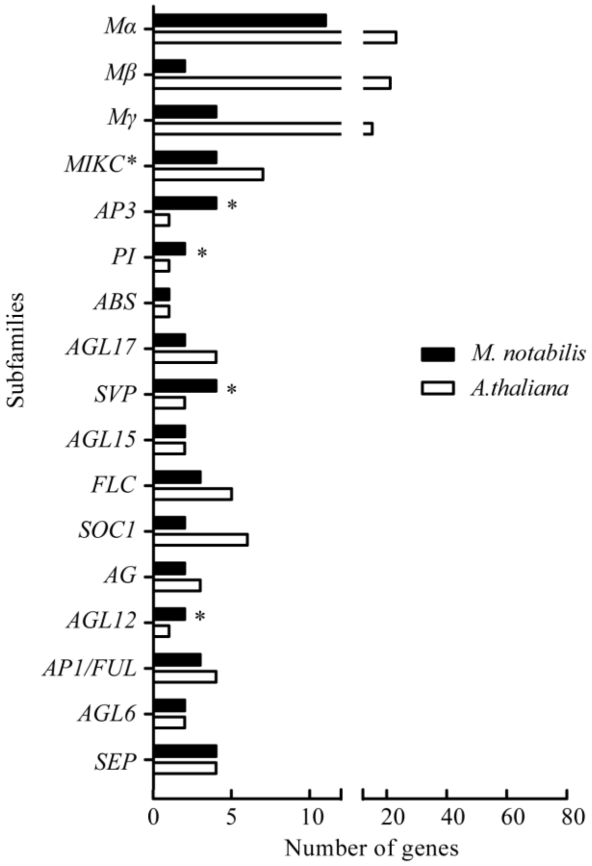

Supplementary Figure S2. Number of genes in MADS-box families/subfamilies in mulberry and *A. thaliana*. For most families and subfamilies, there are more *A. thaliana* genes than mulberry genes. An asterisk indicates a class B gene (*AP3* and *PI* subfamilies), *SVP*, and *AGL12* subfamily member.

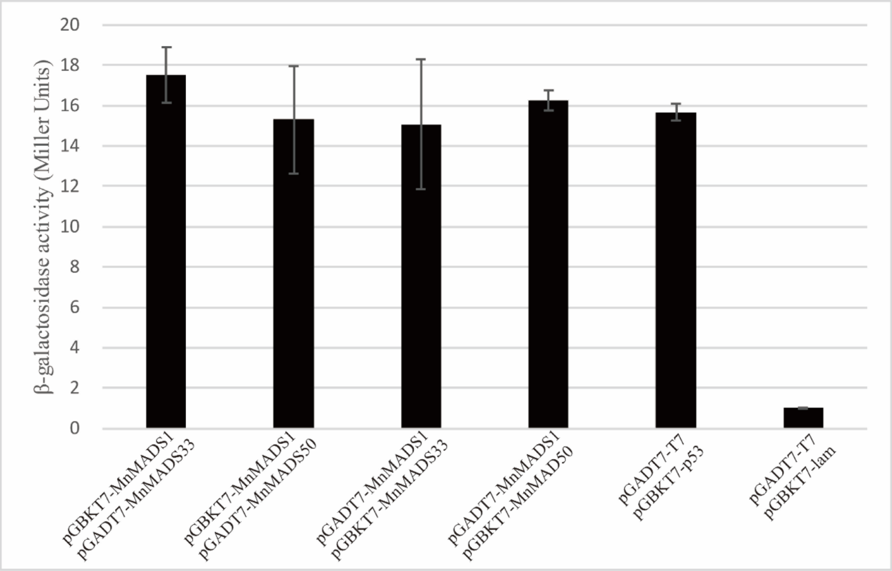

Supplementary Figure S3. β-galactosidase activity analysis. Three biological replicates were performed for the β-galactosidase activity analysis. (n = 3, mean ± measurement range).

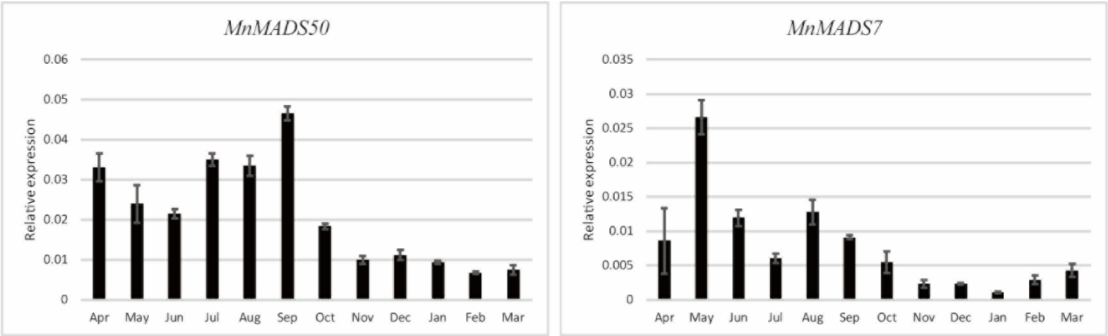

Supplementary Figure S4. Expression analyses of *MnMADS50* and *MnMADS7* in floral buds over one year. Gene expression was measured by qRT-PCR using the *MnCTRL9* as a reference (n = 3, mean ± measurement range).

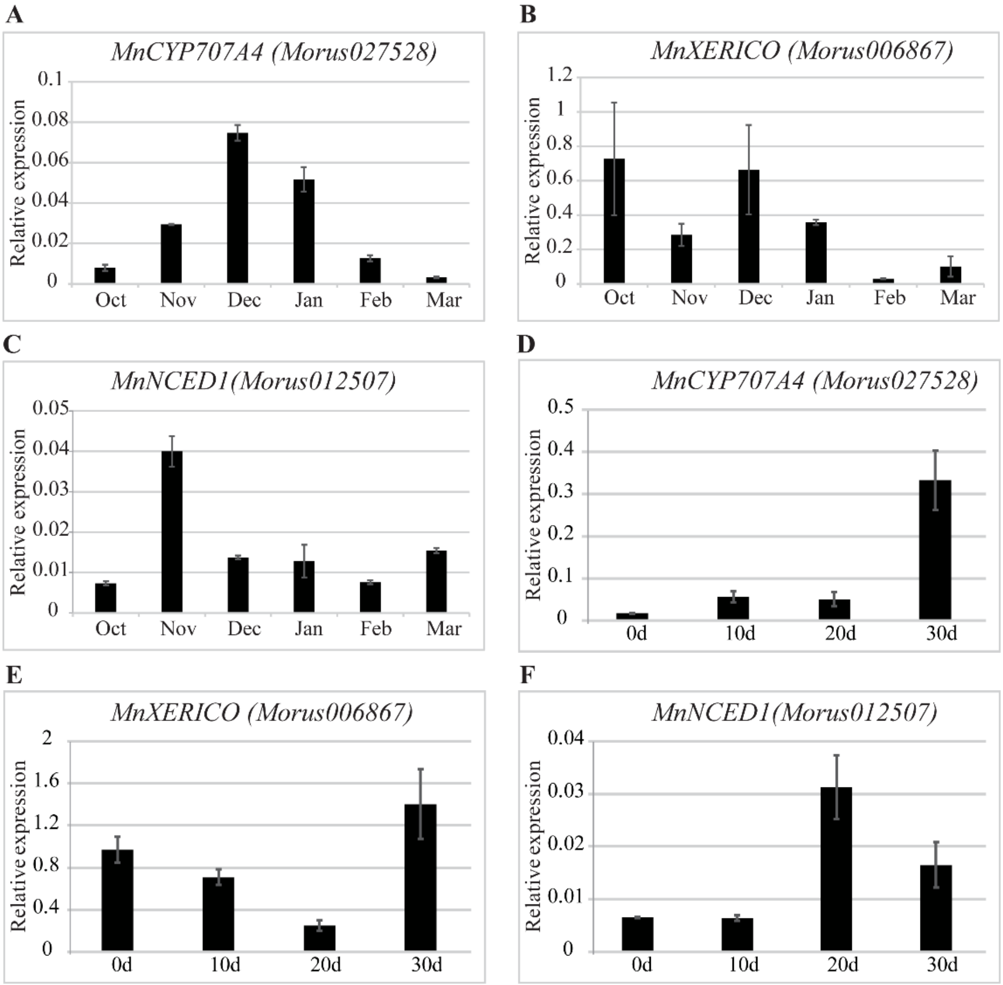

Supplementary Figure S5. Expression analyses of genes related to ABA biosynthesis and catabolism in cold-treated mulberry floral buds under field and artificially controlled conditions. (**A-C**) Relative expressions of *MnCYP707A4*, *MnXERICO*, and *MnNCED1* in floral buds for field sampling during endodormancy. (**D-F**) Relative expressions of *MnCYP707A4*, *MnXERICO*, and *MnNCED1* in floral buds under controlled cold condition. Gene expression was measured by qRT-PCR using the *MnCTRL9* as a reference (n = 3, mean ± measurement range).

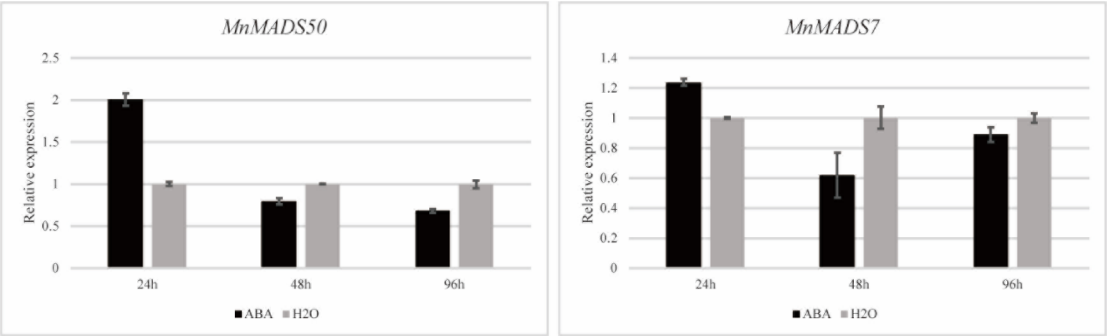

Supplementary Figure S6. Relative expressions of *MnMADS50* and *MnMADS7* in floral buds after treatment for 24, 48, and 96 hours. Gene expression was measured by qRT-PCR using the *MnCTRL9* as a reference (n = 3, mean ± measurement range).

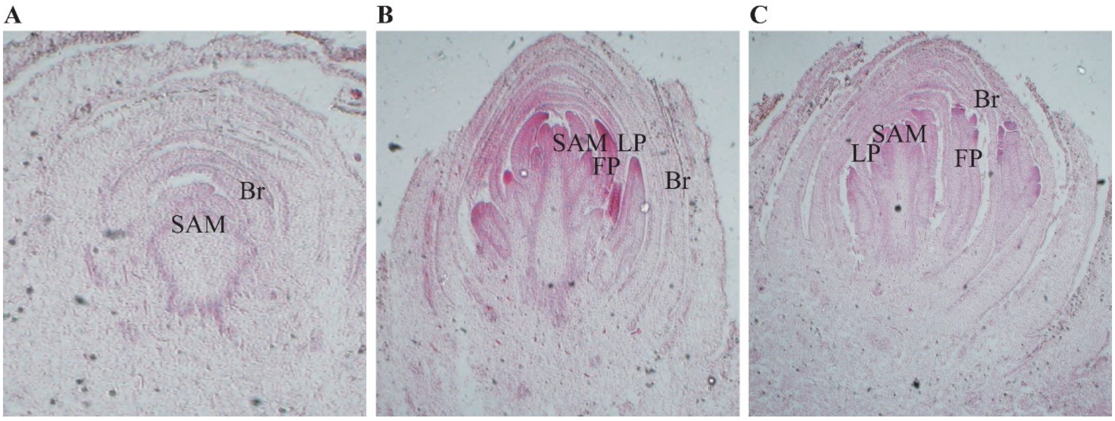

Supplementary Figure S7. Histology of mulberry buds. (**A**) Buds with undifferentiated inflorescence. (**B**) Buds during initial differentiating inflorescence primordia. (**C**) Buds after differentiating inflorescence primordia. *SAM*, *LP*, *Br*, and *FP* indicate the shoot apical meristem, leaf primordia, bract, and flower primordia, respectively.
